# Development of retinoid nuclear receptor pathway antagonists through targeting aldehyde dehydrogenase 1A3

**DOI:** 10.1016/j.isci.2025.113675

**Published:** 2025-10-03

**Authors:** Mark Esposito, Cao Fang, Yong Wei, Alfonso Pozzan, Claudia Beato, Xiaoyang Su, Josiah E. Hutton, Tavis Reed, Xiang Hang, Enrico D. Perini, Wen Wang, Xiaobing Cheng, Yan Pan, Jianshi Yu, Maureen Kane, Malini Manoharan, John Proudfoot, Ileana M. Cristea, Yibin Kang

**Affiliations:** 1 Department of Molecular Biology, Princeton University, Princeton, NJ 08544, USA; 2 Kayothera Inc, Seattle, WA 98102, USA; 3 Ludwig Institute for Cancer Research Princeton Branch, Princeton, NJ 08544, USA; 4 Aptuit (Verona) Srl, an Evotec Company, Verona, Italy; 5 Rutgers University Robert Wood Johnson Medical School, New Brunswick, NJ 08904, USA; 6 WuXi AppTec, Shanghai, China; 7 Department of Pharmaceutical Sciences, University of Maryland, Baltimore, MD 21201, USA; 8 DeepKnomics Labs, Bangalore, India; 9 DiscoveryBytes LLC, Newtown, CT, USA

**Keywords:** Cell biology, Cancer

## Abstract

Aldehyde dehydrogenase 1a3 (ALDH1A3) activity is recognized as a pathogenic trait in cardiometabolic diseases and cancer, though the mechanisms by which ALDH1A3 promotes disease are unclear and effective therapeutic inhibitors of ALDH1A3 are lacking. Whereas the function of the ALDH1A enzymes in development is the conversion of retinaldehyde into all-*trans* retinoic acid (atRA) to activate retinoid nuclear receptor signaling, this pathway is paradoxically hypothesized as a cell-intrinsic tumor suppressor pathway. We resolve this paradox by showing that while ALDH1A3 is overexpressed across diverse cancers, ALDH1A3-expressing tumor cells lose sensitivity to retinoid signaling. Instead, atRA produced by ALDH1A3 acts in a paracrine fashion to activate retinoid nuclear receptor signaling in immune cells to suppress anti-tumor immunity. To inhibit ALDH1A3, we developed a hybrid *in silico* and high-throughput screening approach followed by medicinal optimization to identify first-in-class, oral and safe antagonists of ALDH1A3 with potent anti-tumor immunotherapeutic activity and an optimized drug development profile.

## Highlights

• Tumor ALDH1A3 creates retinoic acid to activate retinoid nuclear receptors in immune cells
• A next-generation hybrid screen identifies potent, selective, and nontoxic ALDH1A3 inhibitors
• Multi-omic studies show that ALDH1A3 functions exclusively to generate retinoic acid in tumors
• Retinoic acid suppresses T cell growth but does not affect ALDH1A3-positive cancers

## Graphical abstract

**Figure:**
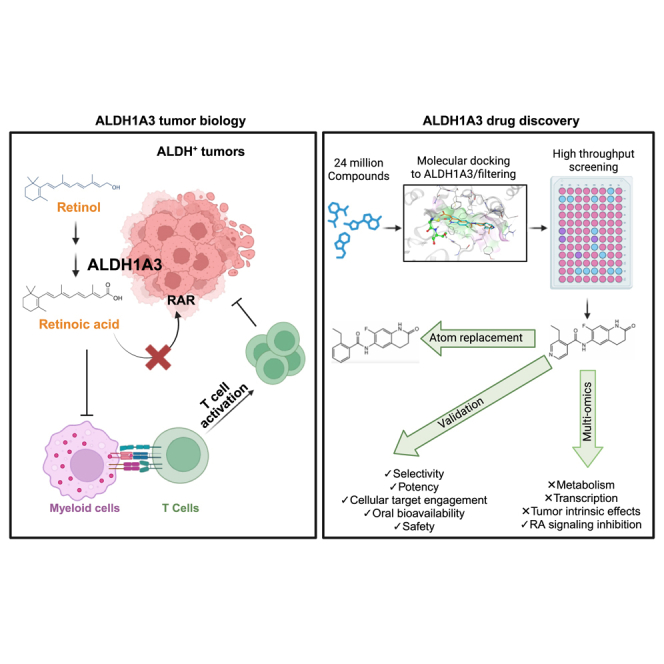

## Introduction

The retinoid nuclear receptor pathway is activated when all-*trans* retinoic acid (atRA), the oxidized metabolite of dietary vitamin A, binds to the retinoid nuclear receptors. This binding results in context-dependent transcriptional regulation, including morphogenic activity in development, regulation of immune tolerance, and metabolic control of lipid metabolism.^1^^,^^2^ In contrast to the well-described role of the retinoid nuclear receptor pathway in normal physiology, its function in disease, particularly cancer, has long remained controversial. Early clinical studies in the 1980s established atRA as a potent therapy in acute promyelocytic leukemia (APL) whose administration leads to deep but short-lived responses. Nearly two decades later, studies showed that this response was due to a unique PML-RARα fusion oncogene in >95% of APL cases that degrades upon binding to atRA, leading to the differentiation of leukemic blasts.^3^

In the two decades between the initial discovery of the clinical benefit of atRA in APL and the mechanistic dissection of the PML-RARα fusion oncoprotein, more than 100,000 patients were enrolled in randomized controlled trials testing various retinoid pathway agonists to prevent cancer. Unexpectedly, retinoid agonism led to equivocal or worse outcomes across these trials, leading to increased rates of solid cancers, increased cardiovascular mortality, and early termination of the trials due to increased all-cause mortality.^4^^,^^5^ Interventional trials testing synthetic retinoid agonists also resulted in worse or equivocal outcomes for patients, particularly in solid cancer patients.^6^ Despite these clinical findings, retinoic acid is often hypothesized as a tumor suppressor owing to its efficacy in treating patients with APL^6^ and its ability to suppress the growth of certain cancer cell lines *in vitro.**^7^*

The aldehyde dehydrogenase 1a isoforms (ALDH1A1, ALDH1A2, and ALDH1A3) are responsible for the final oxidation in the synthesis of retinoic acid from retinaldehyde.^8^ The all-*trans* geometric isomer of retinoic acid is the ligand for the retinoid nuclear receptors (RARα/β/γ) and is preferentially oxidized by ALDH1A2 or ALDH1A3,^9^^,^^10^ while the 9-cis isomer is thought to be formed by ALDH1A1 where it can activate either the RAR or RXR nuclear receptors.^11^ Many studies have independently reported that ALDH1A-expressing cancers are more tumorigenic and predict worse clinical outcomes,^12^ while other studies show that ALDH1A1 and ALDH1A3 promote cardiovascular diseases or obesity.^13^^,^^14^^,^^15^

The description of ALDH1A activity in multiple diseases, including as a pro-tumorigenic enzyme, contrasts with the long-held hypothesis that the retinoid nuclear receptor pathway is tumor suppressive and cardioprotective.^16^ Thus, additional mechanisms have been ascribed to the ALDH1A enzymes such as detoxifying reactive aldehydes, participation in central glycolysis, or oxidative metabolism of chemotherapeutic agents. Here, we utilized genetic approaches to assess the importance of ALDH1A enzymes to atRA formation in human cancers and show that ALDH1A3 is specifically enriched in certain human cancers to create atRA. We utilize next-generation drug discovery techniques to profile the known molecular space for drug-like ALDH1A3 antagonists and advance one key chemotype with low nanomolar inhibitory activity through medicinal chemistry and pharmacology studies. Using these selective inhibitors as a unique research tool, we show that ALDH1A3 oxidizes all-*trans* retinaldehyde into atRA in cancer cells that works as a paracrine factor to suppress anti-tumor T cell immunity. Our development of potent, oral, reversible ALDH1A3 inhibitors overcomes the prior undruggability of the retinoid nuclear receptor pathway and offers a distinct therapeutic approach to tumor immunotherapy and cardiovascular disease.

## Results

### ALDH1A3 activity and retinoid sensitivity are mutually exclusive in tumor cells

**Figure 1:**
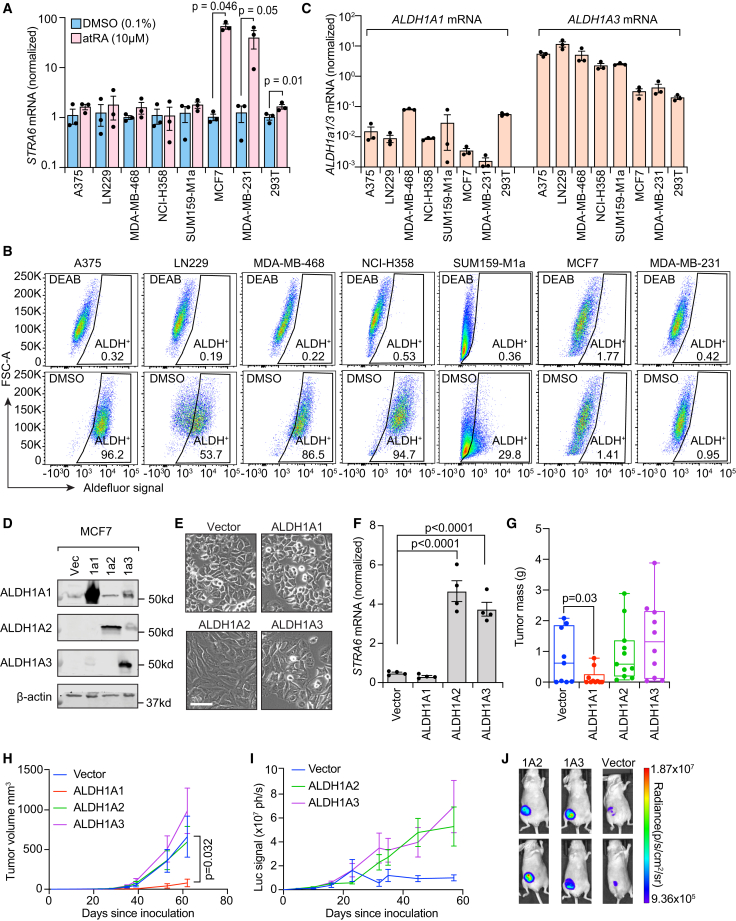
ALDH1A3 is expressed by tumor cells to generate retinoic acid (A) Cancer cell lines were treated with atRA (10 μM) or DMSO (0.1%) for 24 h followed by RNA extraction and RT-qPCR for *STRA6*, a marker of retinoid signaling. *STRA6* mRNA was first normalized to *GAPDH* mRNA (*n* = 3 biological replicates) and then to a universal human reference RNA. Student’s *t* test. Data are represented as mean ± SEM. (B) The Aldefluor assay was conducted on each cell line in the presence of 1 mM DEAB, as the negative control gate, or 1% DMSO. Data representative of *n* > 8 independent experiments. (C) RNA samples from DMSO-treated cells in (A) were assessed by RT-qPCR for *ALDH1A1* and *ALDH1A3* mRNA, which were first normalized to *GAPDH* mRNA (*n* = 3 biological replicates) and then to a universal human reference RNA. Data are represented as mean ± SEM. (D) Human ALDH1A1, ALDH1A2, and ALDH1A3 were ectopically expressed in MCF7 cells as assessed by western blot. (E) Phase-contrast imaging of MCF7 cells stably expressing either ALDH1A1, ALDH1A2, ALDH1A3, or vector control after 10 passages. Image representative of >10 image fields across three separate stable cell derivations. Scale bar represents 150 μm. (F) RT-qPCR analysis of *STRA6* mRNA in MCF7 cell lines with ectopic expression of each ALDH1A isoform compared to vector control cells. *n* = 4 biological replicates, Student’s *t* test. Data are represented as mean ± SEM. (G and H) The SCP28 breast cancer cell line was stably transduced with each ALDH1A isoform and implanted in the mammary fat pad of Nu/Nu mice. Tumor progression was followed by weekly caliper measurement (H) or tumor mass measurement at endpoint (G). *n* = 10 (ALDH1A1 and ALDH1A3), 11(ALDH1A2), and 9 (vector) mice/group. Student’s *t* test. Data are represented as mean ± SEM. (I) SCP28 cells were stably transduced with a retinoid-responsive bioluminescence reporter followed by stable transduction of plasmids expressing *ALDH1A2*, *ALDH1A3*, or empty vector. Weekly intravital imaging was performed to assess *in vivo* retinoid activation. *n* = 12 (ALDH1A2), 11 (ALDH1A3), and 5 (vector) mice/group. Data are represented as mean ± SEM. (J) Representative luciferase images from (I).

*In vitro* studies have shown that atRA can activate canonical retinoid nuclear receptor signaling to suppress tumor growth; however, this response is highly variable and only occurs in a small number of cell lines.^7^^,^^17^^,^^18^ We hypothesized that aldehyde dehydrogenase 1a3 (ALDH1A3) expression should predict this difference in atRA responsiveness as ALDH1A3 expression would suggest the existence of an intact retinoid signaling pathway. Therefore, we selected a panel of cell lines from the Cancer Cell Line Encyclopedia that are ALDH1A3-positive or ALDH1A-negative by RNA sequencing^19^ and treated each line with 10 μM atRA, a supraphysiologic dose that is used in tissue culture to activate retinoid signaling. We assessed retinoid nuclear receptor activation through quantitative reverse-transcription PCR (RT-qPCR) of *stimulated by retinoic acid-6* (*STRA6*) mRNA after a 24 h treatment. *STRA6* encodes a membrane transporter of retinol that is induced by atRA.^20^ Similar to prior studies, we found the response to be variable, with the ALDH1A3-positive cell lines showing no *STRA6* induction, including A375 (melanoma), LN229 (glioblastoma), MDA-MB-468 (breast cancer), NCI-H358 (lung cancer), and SUM159-M1a (breast cancer), while ALDH1A-negative cell lines, including MCF7 and MDA-MB-231 (breast cancer), showed more than 10-fold increases in *STRA6* transcription upon atRA treatment (Figure 1A). To confirm that *STRA6* accurately reported atRA sensitivity, we tested an orthogonal method wherein a retinoic acid response element-firefly luciferase reporter (RARE-Luc) was introduced into both MDA-MB-231 cells and a single-cell subclone of MDA-MB-231, SCP28.^21^ Treatment of either stable cell line with a dose titration of atRA demonstrated a dose-dependent activation of luciferase that saturated at 10 μM atRA (Figure S1A), confirming that *STRA6* mRNA faithfully reported retinoid nuclear receptor signaling in response to atRA. Conversely, we introduced the same retinoid reporter into the ALDH1A3-positive MDA-MB-468 cells, and, as predicted by the *STRA6* mRNA data, treatment with atRA did not increase the low background level of reporter activity (Figure S1A).

To further investigate this finding, we tested if ALDH1A3 was the only enzymatically active aldehyde dehydrogenase (ALDH) in the retinoid-insensitive cell lines. We first tested each cell line with the Aldefluor assay, which assesses intracellular ALDH enzymatic activity. Aldefluor profiling with DMSO or the negative control 4-diethylaminobenzaldehyde (DEAB) confirmed that atRA-insensitive cells (A375, LN229, NCI-H358, MDA-MB-468, and SUM159-M1a) possess high levels of ALDH activity while the sensitive lines (MDA-MB-231 and MCF7) were negative for ALDH activity (Figure 1B). As DEAB is a pan-ALDH inhibitor and thus cannot differentiate between ALDH isoforms, we used RT-qPCR to determine which ALDH1A isoform was expressed. Results demonstrated that *ALDH1A3* expression correlated to Aldefluor activity and inversely correlated with atRA sensitivity. Meanwhile, *ALDH1A1* expression was minimal and uncorrelated to Aldefluor activity (Figure 1C), while ALDH1A2 was not detectable in any cell line (data not shown).

These data suggest that ALDH1A3-positive cancers are retinoid insensitive and thus ALDH1A3 may either possess a moonlighting function in cancer, such as detoxifying reactive aldehydes or controlling glycolysis flux,^22^^,^^23^ or may generate atRA that acts in a paracrine fashion, as has been more recently described.^24^ Myriad studies have hypothesized that ALDH1A3 works in a tumor-intrinsic, growth-promoting role whose inhibition would prevent tumor growth and thus offer a cytotoxic therapeutic strategy.^25^ To test for a tumor-intrinsic role for ALDH1A3, we generated two independent CRISPR-Cas9 knockouts of ALDH1A3 in the cell lines with high endogenous ALDH1A3 activity: MDA-MB-468 (triple-negative breast cancer), A375 (melanoma), and LN229 (glioblastoma). Stable selection of the CRISPRed cell lines demonstrated that ALDH1A3 knockout cells exhibited complete loss of Aldefluor activity while the control gRNA cells exhibited similar Aldefluor activity to parent cells (Figures S1B–S1D). Next, each stable cell line was injected into immune compromised NSG mice either orthotopically (MDA-MB-468 and A375) or subcutaneously (LN229). Tumor growth measurements demonstrated that ALDH1A3 knockout had a minimal effect on primary tumor growth in each cancer type, with replicates showing low variability across repeated experiments (Figures S1E–S1G). These data suggest that while ALDH1A3 is overexpressed in many cancer types, it is not required for tumorigenesis or primary tumor growth in immunocompromised mice.

### ALDH1A3 generates atRA in human cancers and contributes to immune resistance of tumors *in vivo*

These data suggest that ALDH1A3 does not affect cell-autonomous signaling in human cancers that express ALDH1A3, thus suggesting that it may instead generate atRA for paracrine secretion as was recently described in mouse sarcoma.^24^ To first test that ALDH1A3 can generate atRA in tumor cell lines, we took advantage of the finding that the ALDH1A3-negative cell line MCF7 was sensitive to atRA (Figure 1A), and therefore, we ectopically expressed ALDH1A3 along with the related isoforms ALDH1A1 and ALDH1A2 to create stable MCF7 cell lines (Figure 1D). Interestingly, ALDH1A2 and ALDH1A3 expression transformed MCF7 cells from a cuboidal, epithelial-like morphology to a fibroblast-like morphology with poorly demarcated borders. This morphological change was not observed in the ALDH1A1 or vector-expressing cells (Figure 1E). Quantification of retinoid signaling by RT-qPCR of *STRA6* mRNA revealed that the change in morphology in ALDH1A2/1A3-expressing cells was accompanied by an increase in *STRA6* mRNA, indicating that both enzymes produced atRA (Figure 1F) and replicating the published finding that atRA induces morphogenic changes in MCF7 cells.^7^

We next tested if ALDH1A3 generated atRA in tumors growing *in vivo.* Here, we utilized the MDA-MB-231:SCP28 RARE-Luc retinoid reporter clone that showed the strongest reporter activity in response to atRA (Figure S1A) to measure *in vivo* activation of the retinoid reporter by ALDH1A isoforms. Each ALDH1A isoform was stably expressed in these SCP28-RARE-Luc reporter cells, and tumor growth measurements from orthotopically implanted tumors in immunodeficient NSG mice revealed that overexpression of ALDH1A2 or ALDH1A3 did not affect tumor growth while ALDH1A1 suppressed primary tumor growth (Figures 1G and 1H). Luciferase imaging of the RARE-Luc reporter then revealed that both ALDH1A2 and ALDH1A3 synthesize atRA in tumor cells growing *in vivo* (Figures 1I and 1J). Taken together, these data suggest that ALDH1A2 and ALDH1A3 generate atRA in human cancer cells and primary tumors, but this does not appear to affect tumorigenesis or growth in immunodeficient mice.

**Figure 2:**
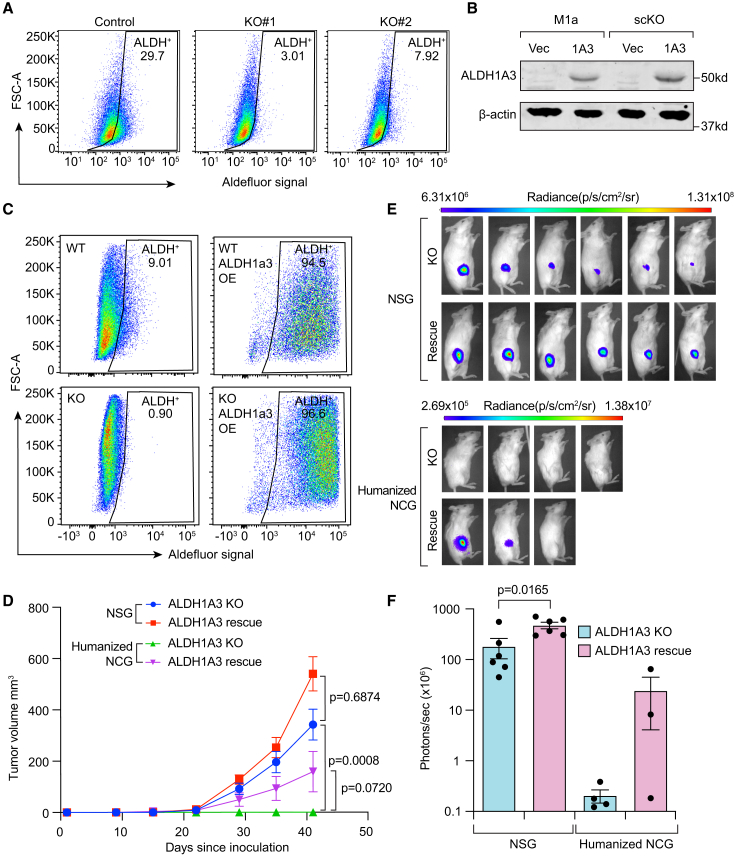
ALDH1A3 is necessary for tumor growth in humanized mice but not immune-deficient mice (A) SUM159-M1a cells were transiently transfected with a plasmid encoding GFP, Cas9, and ALDH1A3-targeting or scrambled guide RNAs followed by sorting for GFP+ cells. The populations were profiled by Aldefluor at passage 5 post sort. (B) Single-cell colonies isolated from (A) were profiled by Sanger sequencing of the *ALDH1A3* locus in the genomic DNA. An ALDH1A3 knockout clone along with the parental population was stably transduced with ALDH1A3 or control expression plasmids. Cell populations were profiled for ALDH1A3 expression and β-actin by western blot. (C) Cells from (B) were tested by the Aldefluor assay. (D) Cells from (B) and (C) were implanted into the mammary fat pad of female NSG and CD34^+^ humanized NCG mice validated for >80% CD45^+^ humanization by Charles River. Tumor growth was measured by weekly caliper measurement. *N* = 6 mice per arm in NSG mice and *n* = 3 or 4 mice per arm in CD34^+^ humanized NCG arms. Statistics by two-way ANOVA. No tumors initiated in the ALDH1A3 knockout arm in the humanized mice. Data are represented as mean ± SEM. (E and F) Bioluminescent quantification at the final measurement day graphed (F) and displayed as representative images (E). Data from (F) are represented as mean ± SEM.

Recent data from Devalaraja et al. suggest that either ALDH1A1 or ALDH1A3 is induced *in vivo* to synthesize atRA that suppresses anti-tumor immunity in a paracrine manner in an immunocompetent mouse sarcoma model.^24^ Here, we assessed if this immunological function of ALDH1A3 is preserved in human cancers by replicating this mechanistic approach in a human solid cancer model using CD34-humanized mice. Initial evaluation of multiple ALDH1A3-positive cell lines showed that the SUM159-M1a cell derivative^26^ preserves both aggressive tumor formation and ALDH1A3 positivity following clonal selection. We next generated ALDH1A3 knockout and rescue SUM159-M1a cell lines through a two-step CRISPR-Cas9 process, first confirming ALDH1A3 activity loss by Aldefluor and genomic knockout by Sanger sequencing. A confirmed single-cell knockout clone was then stably transduced with ALDH1A3 or vector to confirm functional rescue (Figures 2A–2C). This approach ensured that ALDH1A3 expression was the sole variable in this clonal cell background. These cells were orthotopically implanted into fully immune compromised NSG mice or irradiated/human CD34^+^ donor cell-transplanted humanized NCG mice that were confirmed to have >80% humanization by hCD45 profiling. Tumor growth measurements demonstrated that ALDH1A3 knockout minimally reduced tumor growth in the immune compromised NSG mice compared to rescue while ALDH1A3 knockout led to a robust difference in the CD34-humanized mice (Figures 2D–2F). These results support the hypothesis that ALDH1A3 expressed in human cancers suppresses anti-tumor immunity through a paracrine secretion of atRA and validate ALDH1A3 as a potential therapeutic target.

### Development of a hybrid computational drug discovery screen identifies first-in-class ALDH1A3-specific inhibitors

These data identify ALDH1A3 (and potentially ALDH1A2) as a potential target to inhibit retinoid nuclear receptor-mediated immune suppression in cancers, thus offering an orthogonal approach to immune checkpoint blockade in cancer immunotherapy. There has been considerable interest in generating potent ALDH1A antagonists given the prevalent involvement of ALDH1A enzymes across cancer and cardiometabolic disease, yet no specific, drug-like antagonists have been described to date (compounds comprehensively reviewed here^27^). Many non-specific covalent inhibitors of ALDH1A3 have been described, including the cytotoxic *in vitro* tool compound DEAB, as well as compounds that have been tested in humans, including the alcohol aversion compound disulfiram (Antabuse) that has been used clinically to inhibit ALDH2, the bisdiamine compound Win18446 that was tested in healthy volunteers, and dimethyl ampal thiolester (DIMATE), which is under development as a nanoencapsulated cancer therapeutic (ABD-3001) and is reported in phase 1 trials.^6^ In addition, several competitive inhibitors of the ALDH1A isoforms have also been described, including MCI-INI-3, CM037, NCT-501, and CM-010. Lastly, a compound named GA11 has been reported with picomolar cytotoxic activity against ALDH1A3-positive cells in culture, potentially through reversible ALDH1A3 inhibition; however, biochemical activity was not assessed.^28^ While some additional ALDH inhibitors have also been recently described, these are well characterized as selective for ALDH1A1/ALDH2 with minimal potency against ALDH1A2 or ALDH1A3.^29^

To assess whether any of these reported ALDH1A inhibitors are viable starting points for medicinal chemistry, we first assessed their potency and selectivity in biochemical assays. We adapted a well-established *in vitro* assay to measure ALDH1A enzymatic activity and titrated known substrates (e.g., propanaldehyde) or cofactor (e.g., NAD+) concentrations to optimize reaction conditions for recombinant ALDH1A1, ALDH1A2, and ALDH1A3 (Figures S2A and S2B). We then tested each published ALDH inhibitor against the three human ALDH1A enzymes to assess inhibitory activity and isozyme selectivity. Results demonstrated that among the covalent inhibitors, disulfiram, WIN18446, and DEAB were strong ALDH1A1 inhibitors, moderate against ALDH1A2 and weak against ALDH1A3. Interestingly, DIMATE did not show any activity against any of the enzymes despite its entry into human trials (Figure S2C). Among the reversible inhibitors, only NCT-501 showed activity against ALDH1A1 while MCI-INI-3, GA11, CM037, and CM010 showed no activity up to 10 μM (Figure S2C). DIMATE is reported to be metabolized within cells, which then leads to inhibition of ALDH enzymes^30^; thus, we tested DIMATE in the cell-based Aldefluor assay on both HepG2 cells that express ALDH1A1 and A375 cells that express ALDH1A3. Results demonstrated that DIMATE showed no dose-dependent inhibition of activity in A375 cells while it showed a 13% inhibition of Aldefluor activity in HepG2 cells at the top dose of 10 μM following a 45 min incubation at 37°C (Figure S2D). Thus, among all the inhibitors tested, only WIN18446, disulfiram, NCT-501, and DEAB showed an ability to inhibit ALDH1A activity at a therapeutically relevant concentration of 10 μM, and only WIN18446 or DEAB showed activity in the cellular Aldefluor assay using ALDH1A3-positive A375 cells (Figure S2F). Attempts have been made to optimize both the DEAB and WIN18446 structures to improve drug-likeness, yet these have failed to produce therapeutically viable compounds.^31^^,^^32^

**Figure 3:**
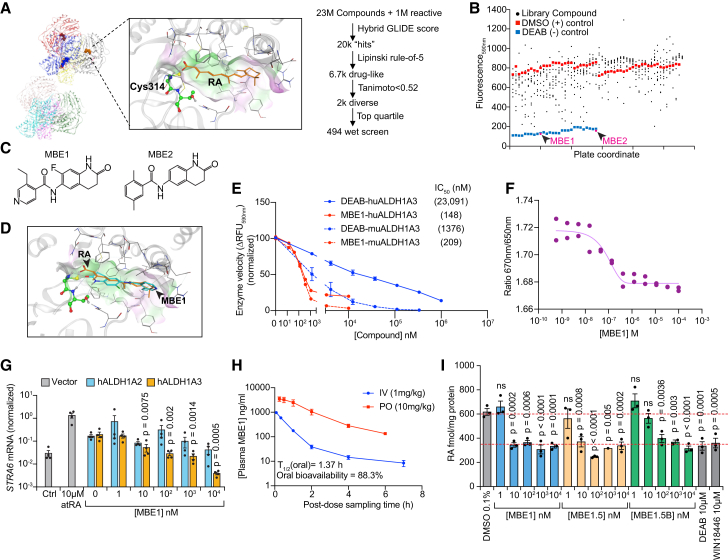
Identification of potent ALDH1A3 inhibitors through hybrid high-throughput screening workflow (A) The virtual screening workflow for virtual docking campaign first generated a constrained docking pose for the ALDH1A3 active site bound to atRA. Catalytic residue cysteine 314 and atRA are highlighted in the structure (left). A collection of 24 million compounds was computationally docked in this pose to provide a hybrid GLIDE score. “Hit” compounds were filtered according to the binding, drug-likeness, and diversity criteria (right). (B) A high-throughput method of quantifying ALDH1A3 kinetic activity was optimized for 96-well format. A screening library of 494 candidate molecules with randomly allocated DMSO (0.1%) or DEAB (1 mM) wells was then tested. Compounds exhibiting inhibition within 2-fold the potency of DEAB-negative controls were confirmed in secondary screening. Data points for hit compounds MBE1 and MBE2 are highlighted with arrowheads. (C) Positive hits from (B), including MBE1 and MBE2, are shown. (D) The structure of hit compound MBE1 was superimposed on the atRA docking pose in the human ALDH1A3 crystal structure from (A). (E) Dose titration of MBE1 and DEAB performed against both human and mouse ALDH1A3 enzymes using the adapted biochemical assay. *n* = 3 biological replicates per concentration. Data are represented as mean ± SEM. IC_50_ values calculated by 4-parameter equation. (F) An MST assay developed with human ALDH1A3 was used to determine the K_D_ for MBE1 with ALDH1A3. (G) MCF7 cells engineered to stably express ALDH1A2 or ALDH1A3 were cultured with 0.1% DMSO (negative control), 10 μM atRA (positive control), or a dose titration of MBE1. After 24 h, RT-qPCR for *STRA6* was performed and was normalized to *GAPDH* levels. *n* = 4 biological replicates. One-Way ANOVA, *p* values indicate the statistical tests comparing indicated groups to untreated group. Data are represented as mean ± SEM. (H) MBE1 was formulated into a solution for intravenous (i.v.) dosing or a suspension for PO dosing and administered to fasted male CD1 mice at 1 mg/kg i.v. and 10 mg/kg oral gavage (*per os*; PO). Plasma was analyzed at each time points for MBE1 concentration by high-performance liquid chromatography. Half-life, bioavailability, and exposures were calculated by WinNonLin software. *n* = 3 biological replicates. Values at 24 h were below lowest level of quantitation. Data are represented as mean ± SEM. (I) Quantification of atRA in MDA-MB-468 cells treated with each compound for 24 h. *n* = 3 biological replicates. Student’s *t* test. Data are represented as mean ± SEM.

Given that none of the currently reported ALDH1A inhibitors are viable starting points for medicinal chemistry optimization, we sought to take a distinct approach toward identifying novel, potent, and specific ALDH1A3 inhibitors. We therefore developed a next-generation drug discovery approach to computationally profile a large region of the known molecular space for ALDH1A3-interacting compounds. We adapted an *in silico* docking screen using a substrate-constrained docking pose of ALDH1A3 derived from the crystal structure of ALDH1A3 bound to atRA.^33^ We biased the screening away from ALDH1A1 or ALDH3A1 docking by substituting and then counterscreening against unique residues present in the active site of either enzyme (Figure 3A). A screening collection of 23 million compounds plus an additional 1 million reactive compounds was then filtered using this hybrid GLIDE scoring system to probe the potential for residency of each compound in the constrained active site. To optimize for drug-like compounds, rule-of-five violating compounds were deprioritized, while higher functional diversity by Tanimoto scores was prioritized to emphasize diversity of our focused hit library. The result was a series of 494 high diversity hit molecules that were formatted for biochemical screening (Figure 3A, right).

To internally control the screening assay, uniformly distributed DMSO wells were used as negative controls, and 1 mM DEAB was used as a positive screening control. Compounds were screened with this system using a cutoff value of two times the average DEAB signal. The screening identified 8 hit compounds at this threshold, of which two compounds were identified as true positives in confirmatory screening (Figures 3B and 3C). These two leads were named MBE1 and MBE2, and they showed remarkable similarity in molecular structure with a quinolone group that putatively binds to the catalytic cysteine of ALDH1A3 and a nonpolar aromatic ring that mimics the ionone ring of all-*trans* retinaldehyde (Figures 3C and 3D). Confirmatory screening identified that MBE1 was more potent than MBE2 while the structure of MBE2 predicted worse ADME characteristics. Compound MBE1 was superimposed with the constrained docking pose of ALDH1A3 and atRA, demonstrating remarkable alignment between the structures in the ALDH1A3 active site (Figure 3D).

A dose titration of MBE1 in the substrate competition assay against recombinant human and mouse ALDH1A3 demonstrated IC_50_ values of 148 and 209 nM, respectively. This compared favorably to 23,091 and 1,376 nM for DEAB in the same experimental conditions (Figure 3E). MBE1 was next screened for nonspecific cytotoxicity in ALDH1A3^+^ cancer cells (MDA-MB-468) and normal human fibroblastic cells (HaCaT) as compared to other reported ALDH1A inhibitors. Results demonstrated that MBE1 and WIN18446 were nontoxic to the highest tested dose of 10 μM, while GA11 showed potent cytotoxicity above 100 nM (Figures S3A and S3B), demonstrating that the GA11 scaffold has off-target cytotoxicity. We also tested for specificity within the ALDH1 subfamily by assessing biochemical inhibition of each isoform; results demonstrated that MBE1 was highly specific to ALDH1A3 with no inhibition of ALDH1A1 or ALDH2, while it showed a 4-fold lower affinity for ALDH1A2 (Figure S3C). Finally, we developed a microscale thermophoresis (MST) assay with ALDH1A3 using NHS-NT647 as an amine-based fluorescent probe to test the binding of MBE1 to recombinant human ALDH1A3. This method was optimized in a low-salt buffer and indicated a potent K_D_ of 71.7 nM (Figure 3F), demonstrating direct binding of MBE1 to purified ALDH1A3 with a K_D_ value consistent with the biochemical data.

Given the favorable physiochemical predictions and biochemical activity for MBE1 (Table 1), we next sought to determine if it could block retinoid activation in MCF7 cells expressing either ALDH1A2 or ALDH1A3. We performed a 24-h dose titration of MBE1 in these cells compared to atRA at 10 μM or DMSO as controls. Retinoid inhibition by quantification of *STRA6* mRNA by RT-qPCR demonstrated that MBE1 was highly potent with a cellular IC_50_ of 3.67 nM for ALDH1A3. Testing the same conditions in MCF7-ALDH1A2 cells showed that MBE1 was selective for ALDH1A3 given an IC_50_ > 1,000 nM for ALDH1A2 (Figure 3G).

**Table 1:** Physiochemical, biochemical, and metabolic profile of lead compounds MBE1 and MBE1.5

Trait	Assay/note	Optimal threshold	MBE1	MBE1.5	MBE1.5B
**Molecular**					

MW	–	<350	313	312	326
LogP	–	<3.5	2.1	3.1	3.5
H bond donor	–	</ = 2	2	2	2
TPSA	–	<120	71	58	58

**Potency**					

Microscale thermophoresis	biophysical assay	<100 nM	71.7 nM	42.1 nM	1,800 nM
IC_50_ molecular, mouse ALDH1A3	recombinant activity assay	<150 nM	244 nM	149 nM	1,854 nM
IC_50_ molecular, human ALDH1A3	recombinant activity assay	<150 nM	144 nM	83 nM	2,712 nM
EC_50_ cellular, human ALDH1A3	Aldefluor in A375 cells	<30 nM	5.1 nM	1.9 nM	155 nM
EC_50_ cellular, human ALDH1A3	*STRA6* qPCR in MCF7-ALDH1A3 cells	<50 nM	3.67 nM	0.53 nM	ND

**Selectivity**					

Cytotoxicity	48 h tetrazolium proliferation assay	>10 μM	>100 μM	>100 μM	not tested
IC_50_ molecular, mouse ALDH1A2	recombinant activity assay	>1,000 nM	3,333 nM	4,000 nM	not tested
IC_50_ molecular, human ALDH1A2	recombinant activity assay	>1,000 nM	3,333 nM	5,000 nM	not tested
EC_50_ cellular, human ALDH1A2	*STRA6* qPCR in MCF7-ALDH1A2 cells	>50 nM	1,666 nM	89 nM	not tested

***In vitro* ADME**					

Kinetic solubility pH 7.6	standard ADME panel	>30 μM	>200 μM	33.3 μM	not tested
Kinetic solubility pH 4.5 (μM)	standard ADME panel	>30 μM	>200 μM	34.2 μM	not tested
MDCK-MDR2 permeability A to B	standard ADME panel	high	high	high	not tested
MDCK-MDR2 (B to A/A to B)	standard ADME panel	<3	2.42	0.6	not tested
Microsomal T_1/2_ (human)	standard ADME panel	t_1/2_ > 120 min	>145 min	>145 min	not tested
Microsomal T_1/2_ (mouse)	standard ADME panel	t_1/2_ > 100 min	43.6 min	112.4 min	not tested
Hepatocyte T_1/2_ (human)	standard ADME panel	t_1/2_ > 120 min	>216.8 min	>216.8 min	not tested
Plasma stability T_1/2_ (human)	standard ADME panel	>240 min	>289.1 min	–	not tested
Protein binding (human)	standard ADME panel	<95%	47.30%	86.30%	not tested
Cyp1a2 inhibition at 10 μM	standard ADME panel	<50% at 10 μM	8.80%	11.60%	not tested
Cyp2c9 inhibition at 10 μM	standard ADME panel	<50% at 10 μM	13.90%	5.60%	not tested
Cyp2C19 inhibition at 10 μM	standard ADME panel	<50% at 10 μM	15.80%	0%	not tested
Cyp2D6 inhibition at 10 μM	standard ADME panel	<50% at 10 μM	61.30%	0%	not tested
Cyp3a4 inhibition at 10 μM	standard ADME panel	<50% at 10 μM	42.00%	6.30%	not tested
hERG, IC_50_ (patch-clamp)	patch-clamp	>30 μM	26.56 μM	>30 μM	not tested
hERG, IC_50_ (dofetilide binding assay)	standard ADME panel	>30 μM	>30 μM	>30 μM	not tested

To assess the *in vivo* pharmacokinetics of MBE1, we assessed the measured plasma concentrations following a 1 mg/kg intravenous or 10 mg/kg oral dose of MBE1 in fasted male CD-1 mice. Results demonstrated rapid absorption with a maximum plasma concentration (C_max_) between 15 and 30 min, an oral half-life of 1.37 h, high exposures calculated by AUC_0–24h_, and a calculated oral bioavailability of 88.3% (Figure 3H). Given that multiple orthogonal assays demonstrated high potency of MBE1 in blocking ALDH1A3 activity and *in vivo* data showed excellent bioavailability and low to moderate clearance, we tested a standard panel of ADME tests to identify any potential liabilities with the MBE1 structure that may prevent its further medicinal optimization into a drug candidate. These tests showed high solubility, high metabolic stability following human and mouse microsome/hepatocyte/plasma coincubation, high cell membrane permeability/low efflux in the MDCK-MDR2 bidirectional permeability assay, minimal hERG binding as assessed both by patch-clamp and dofetilide competition binding, and minimal activity against the five major cytochrome P450 enzymes, with 10 μM MBE1 only inhibiting CYP2D6 at a moderate level (Table 1). A screen against 44 common, clinically validated off-target proteins that commonly show up in profiling demonstrated that MBE1 only interacted with PDE3α, albeit at >100-fold higher concentrations than its IC_50_ against ALDH1A3 (Tables 1 and 2). These data indicate that MBE1 is a viable ALDH1A3 inhibitor across four orthogonal inhibition assays and that it has favorable properties as a scaffold for continued drug development.

**Table 2:** Off-target profiling of MBE1 activity against common enzymes and transporters with known toxicology liabilities

Target	Reference	Reference dose (nM)	Inhibition by reference at listed dose (%)	Inhibition by MBE1 at 10 μM (%)
Alpha2A	yohimbine	1,000	96.99	−1.15
NET	protriptyline	1,000	101.32	−19.93
V1A	[d(CH2)51,Tyr(Me)2]-AVP	100	91.20	5.88
CCKa	CCK-8s	1,000	99.67	−1.45
NMDA	MK801	10,000	100.30	−13.59
Ca2+-L	nitrendipine	100	107.69	−0.09
5HT2B	(±)DOI	10,000	97.38	−6.81
5HTT	imipramine	1,000	98.37	−24.24
DAT	BTCP	1,000	101.51	−13.05
hERG	dofetilide	1,000	100.52	−28.15
5HT2A	ketanserin	1,000	99.23	−16.05
H1	pyrilamine	1,000	99.44	−11.71
M1	pirenzepine	1,000	97.60	−7.68
M2	methoctramine	1,000	94.45	−6.51
M3	4-DAMP	1,000	100.20	−13.45
AR	progesterone	1,000	106.04	−10.12
GR	dexamethasone	100	96.26	−13.48
ADORA2A	CGS 15943	1,000	97.84	−15.67
Alpha1A	WB 4101	100	98.68	−3.34
GABAA	flumazenil	1,000	95.93	−5.08
D1	SCH 23390	1,000	100.71	−16.45
D2	7-OH-DPAT	1,000	100.10	1.45
op-delta	naltrindole	100	103.77	5.54
op-kappa	U-50488	1,000	102.60	−12.62
op-mu	DAMGO	1,000	102.03	3.98
5HT3	MDL 72222	10,000	96.06	−18.62
5HT1B	serotonin	10,000	101.22	5.26
H2	cimetidine	10,000	97.65	4.29
Beta1	atenolol	100,000	107.47	−8.49
Beta2	ICI 118551	1,000	96.99	3.23
nACHR-Alpha7	MLA	1,000	101.20	−9.85
5HT1a	8-OH-DPAT	1,000	96.68	3.78
CB1	CP 55940	100	97.93	−7.11
CB2	WIN 55212-2	1,000	98.51	5.07
ACHE	neostigmine bromide	100,000	99.81	5.81
COX1	diclofenac	5,000	100.72	−2.65
COX2	NS-398	5,000	102.27	−1.05
LCK	staurosporine	200	129.4	−2.81
MAO-A	clorgyline	1,000	99.9	−8.05
PDE3A	trequinsin	50	100.41	75.80
PDE4D2	IBMX	1,000,000	99.945	11.85
Eta agonist	endothelin 1	300	118.83 (activation)	−0.16 (12 μM MBE1)
Eta antagonist	BQ-123	75	99.32	4.57
hNav1.5	tetracaine	10,000	97.11	−0.59
hKCNQ1	chromanol 293B	10,000	44.68	11.89

### Medicinal chemistry identifies a structure-activity relationship for ALDH1A3 inhibitors

To identify a structure-activity relationship (SAR) for ALDH1A3 inhibitors, we first identified compounds with highly similar structures that were commercially available (MBE3.1–3.8) and tested these for biochemical activity against human and mouse ALDH1A3 (Figure S4A). Results demonstrated an exquisite sensitivity to substitutions on the pyridine/phenyl ring, with alkyl substitutions only tolerated ortho to the carboxamide linker in either the phenyl or pyridine rings (such as in MBE3.3 vs. MBE3.6). We next synthesized single atom deletions from the MBE1 structure; SAR from synthesized compounds demonstrated that each functional group of MBE1 was strictly necessary except the 7′ fluoro of the quinolone (MBE1.6) and the pyridine nitrogen (MBE1.5). Next, we tested additional alkyl groups on the phenyl ring of MBE1.5 at the ortho position (MBE1.5A–D); these demonstrated flexibility for nonpolar additions to MBE1.5 but did not improve potency (Figures S4A and S4B) while also introducing potential metabolic liabilities.

To test if the biochemical SAR translated to the cellular context, we profiled compounds MBE1-MBE1.6 in the Aldefluor assay at 100 nM (Figure S4C). Results were consistent with biochemical data by showing the highest activity for MBE1, MBE1.5, and MBE1.6, while MBE3.3 and MBE1.2 showed dramatically lower activity despite differing by only 1–2 carbons from MBE1.6 or MBE1.5. Next, we performed a dose titration of MBE1.5A–D derivatives in cells given the exceptionally tight SAR around the ortho-substituted alkyl group as demonstrated by the low activity of MBE1.5B (Figure S4B). A dose titration in the Aldefluor assay showed that MBE1.5B was far less potent than MBE1.5A, C, or D, confirming that both the biochemical and Aldefluor assays reported the same SAR (Figure S4D). This loss of activity for MBE1.5B in the cellular Aldefluor and biochemical assays, despite differing from MBE1.5 by a single carbon (Table 1), demonstrates the exquisite sensitivity for substitutions in this area of the molecule and provides an important site for additional SAR. This scaffold has been expanded in a subsequent medicinal chemistry effort with >200 compounds annotated with ALDH1A3 activity.^34^^,^^35^

### Intensive target engagement validation verifies MBE1 scaffolds as *bona fide* ALDH1A3 inhibitors

Given the development of a clear SAR for the MBE1 scaffold interaction with ALDH1A3 as well as the promising solubility, metabolism, and pharmacokinetic profile, we next sought to intensively validate MBE1 derivatives in biophysical, biochemical, and cellular target engagement assays as well as pharmacokinetic and disease models. To test the biophysical interaction of MBE1.5 with ALDH1A3, MST demonstrated higher affinity than MBE1 with a K_D_ of 42.1 nM (Figure S4E), while the low activity analog, MBE1.5B, yielded a K_D_ of 1,800 nM. We next developed a nanoscale differential scanning fluorimetry (DSF) assay for ALDH1A3-interacting compounds. The results showed that MBE1.5 led to >8.5°C thermal stabilization of purified ALDH1A3 protein at 10 and 100 μM concentrations (Figure S4F). This protocol was validated with MBE1, which showed similar stabilization, and MBE1.5B, which showed little stabilization (data not shown). As a key test of retinoid nuclear receptor pathway inhibition, we tested the ability of MBE1, MBE1.5, and MBE1.5B to reduce the production of atRA by endogenous ALDH1A3. A dose titration of each compound compared to the positive controls DEAB and WIN18446 demonstrated that MBE1 and MBE1.5 potently reduced atRA formation in MDA-MB-468 cells while MBE1.5B exhibited a higher IC_50_ (Figure 3I), thus confirming that MBE1 and related compounds block ALDH1A3 in cells and that ALDH1A3 produces atRA in retinoid-insensitive human cancer cells.

We next performed an expanded series of biochemical assays to determine MBE1.5 selectivity and mechanism of action. In selectivity profiling using recombinant enzymes for ALDH1A1, ALDH1A2, ALDH2, ALDH1L1/1L2, and ALDH3A1, MBE1.5 was highly selective for ALDH1A3, with minimal inhibition of ALDH1A2 and no inhibition of other tested ALDH enzymes (Figure S4G; Table 1). We next assessed the competition mode for MBE1.5, assuming that it would behave as a competitive inhibitor given that the docking pose screened for compounds that bind the active site. Rather, we found that MBE1.5 behaved as a noncompetitive inhibitor of both the cofactor and substrate (Figure S4H), potentially due to bridging of both the substrate and cofactor binding sites. Transforming the data into double reciprocal plots indicates that the ALDH1A3 enzyme shows cooperativity and thus the inhibitors described here demonstrate a non-classical competition mode (Figure S4H, right).

Next, we developed three orthogonal methods to assess MBE1.5 selectivity and potency in cellular assays, as these cellular assays provide a more physiologically relevant method to assess target engagement and specificity. In the first method, we ectopically expressed ALDH1A1, ALDH1A2, ALDH1A3, or vector control in both MCF7 wild-type and SUM159-M1a-ALDH1A3 knockout cells. We confirmed expression of each enzyme and then performed a dose titration of the Aldefluor assay in each cell derivative with MBE1.5. Results demonstrated that MBE1.5 shows high potency in inhibiting ALDH1A3 but not ALDH1A1 or ALDH1A2 in the Aldefluor assay (Figure S5A). Given that MBE1.5 thermally stabilizes recombinant ALDH1A3 by DSF, we next sought to demonstrate direct binding and thermal stabilization of endogenously expressed ALDH1A3 in cultured cells. Therefore, we performed thermal proteome profiling (TPP) on A375 melanoma cells exposed to DMSO, 100 nM MBE1, and 1 μM MBE1. Notably, MBE1 was used instead of MBE1.5 due to its lower plasma protein binding coefficient (Table 1). These two doses would be expected to saturate ALDH1A3 binding based on the biochemical affinity while the high dose may be expected to identify potential MBE1 off-targets. Following data quality control, more than 8,000 proteins were identified in the proteomic data across the three replicates, which gave rise to 6,853 proteins with no more than 3 missing values across all temperatures or that had data for only 5 lowest melting temperatures. We next assessed for melting point changes in the dataset using a 1.5°C cutoff and *p* < 0.05; 112 proteins were identified in the comparison between 100 nM MBE1 and DMSO, including ALDH1A3. ALDH1A3 was also similarly stabilized by MBE1 treatment at 1 μM (Figure S5B). ALDH1A3 demonstrated a classic sigmoidal melting curve in contrast to many of the other identified proteins that exhibited irregular melt curves. To focus on proteins with robust melting curves, we selected only proteins demonstrating a coefficient of variance <0.1 in both the 100 nM vs. DMSO and 1 μM vs. DMSO condition. This filtering resulted in only two remaining potential MBE1 interaction targets in cells, neuroplastin (NPTN) and ALDH1A3 (Figure S5C). To confirm whether ALDH1A3 or NPTN are *bona fide* MBE1-interaction targets, we performed a cellular thermal shift assay (CETSA) followed by western blot using cells treated with 100 nM MBE1 or 0.1% DMSO. Consistent with the TPP analysis, ALDH1A3 bound to MBE1 showed increased stability and solubility at high temperatures (Figure S5D). In contrast, NPTN was not stabilized by MBE1 (Figure S5D), suggesting that NPTN was a false-positive hit generated from the TPP experiment. Interestingly, the thermal stabilization temperature was different between TPP and CETSA studies, suggesting that the detergents used in TPP may lower the stabilization temperature of ALDH1A3 compared to the physiologic conditions during CETSA. Finally, we validated that MBE1.5 blocked the physiological function of ALDH1A3 by testing a dose titration of MBE1.5 in the retinoid inhibition assay using MCF7-ALDH1A3 cell-based readout. This demonstrated that MBE1.5 was exceptionally potent, exhibiting an IC_50_ of less than 1 nM (Figure S5E).

We next profiled MBE1.5 for a variety of ADME characteristics, which showed improved or similar properties to MBE1 in all variables except decreased kinetic solubility; alternately, MBE1.5 showed improved profiles for CYP2D6 inhibition due to the substitution of a phenyl for the pyridine (Table 1). Next, we assessed *in vivo* pharmacokinetics for MBE1.5; data showed improved half-life but reduced bioavailability (Figure S5F). In order to accommodate *in vivo* dosing and to also determine any potential toxic effects of MBE1.5, we performed a 7-day repeat-dose study administering a suspension of MBE1.5 at 50 mg/kg twice daily via oral gavage. Body weight was monitored daily and showed no difference between MBE1.5 and vehicle groups (Figure S5G). At the final day of dosing, a complete blood profile was collected to determine if any changes in hematology were observed in these mice. Results demonstrated no detectable difference in blood profiles between MBE1.5 and vehicle groups (Figure S5H).

Finally, to establish a pharmacokinetic profile throughout dosing, MBE1.5 was analyzed in serum 12 h post dose on day 1 and day 7. Results clearly demonstrated that MBE1.5 accumulated in mice at this high dose of 50 mg/kg twice daily oral gavage (Figure S5I), necessitating a strategy that minimized the risk of accumulation. To accommodate consistent plasma concentrations without accumulation, MBE1.5 was formulated to a lower concentration in mouse diet that would administer a dose equivalent to 25 mg/kg daily. This formulated diet was provided to mice for 5 days, at which point serum was analyzed for MBE1.5 concentration at 6:30 a.m. and 4:00 p.m. on day 5 to capture differences in mouse feeding cycles. Analysis of MBE1.5 in serum demonstrated a steady-state level between 107 and 84.1 ng/mL, which equates to a nadir of ∼270 nM or more than 100-fold the IC_50_ in the retinoid assay (Figure S5J).

### The MBE1 compounds block ALDH1A3 *in vivo* to reverse atRA-mediated anti-tumor immune suppression

**Figure 4:**
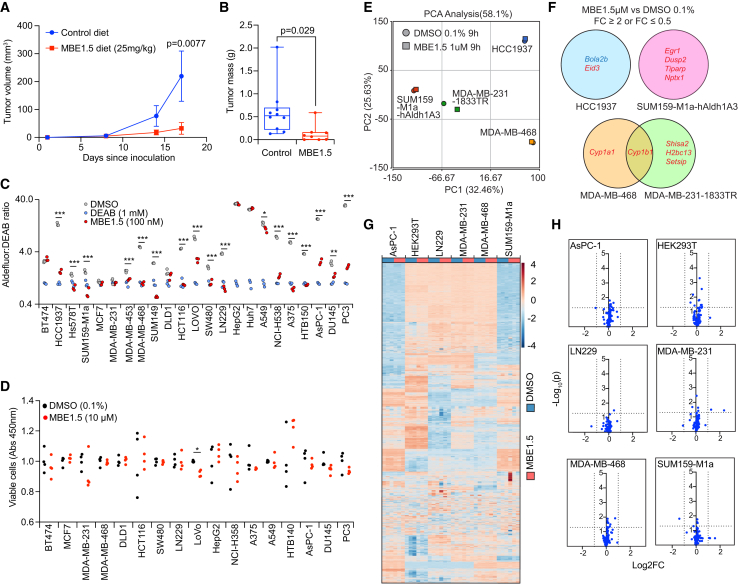
Pharmacologic inhibition of ALDH1A3 prevents tumor growth *in vivo* but does not impact cell survival, transcription, or metabolism in culture (A) MBE1.5 was formulated into standard research mouse food at 270 ppm to yield a daily dose roughly equivalent to 40 mg/kg calculated by average food intake and mouse mass. Male C57BL/6 mice were subcutaneously injected with T3-MCA cells and were randomized to MBE1.5 or control treatment on day 7 to achieve the same mean tumor size across groups. Mouse tumors were measured by weekly caliper measurement. *n* = 10 mice in control group and *n* = 8 mice in MBE1.5 group. Data are represented as mean ± SEM. Mann-Whitney U test. (B) At endpoint, mice from (A) were euthanized and their final tumors removed and weighed. *n* = 8 mice (MBE1.5) and 10 mice (control). Data are represented as mean ± SEM. Student’s *t* test. (C) A panel of cancer cell lines was profiled by the Aldefluor assay with 0.1% DMSO (positive control), DEAB (negative control), or MBE1.5 at 100 nM. Cell line panel was profiled across *n* = 3 independent experiments, and values were normalized to DEAB median fluorescent intensity within each study. Data are represented as mean ± SEM. Student’s *t* test. ∗∗∗*p* < 0.005, ∗∗*p* < 0.01, ∗*p* < 0.05. (D) A subset of cell lines from (C) were seeded to 96-well plates at predetermined cell densities optimized on a per-cell line basis to support a 48 h proliferation assay. DMSO or MBE1.5 was added to wells, and 48 h proliferation was measured by the WST-8 tetrazolium dye assay. *n* = 4 biological replicates. Data are represented as mean ± SEM. Student’s *t* test. ∗*p* < 0.05. (E and F) Three cells lines positive for ALDH1A3 (SUM159-M1a, HCC1937, and MDA-MB-468) and one cell line negative for ALDH1A3 (MDA-MB-231) were cultured in the presence of MBE1.5 or DMSO for 9 h followed by RNA sequencing analysis. Deconvoluted transcriptomic data for each condition were analysis by principal component analysis (E), and individual transcripts altered by > 2-fold between MBE1.5 and DMSO treatment conditions are listed (F). (G and H) Metabolomic analysis of cell lines treated with DMSO or MBE1.5 for 6 h followed by organic extraction of samples and unbiased metabolomic profiling is displayed as a heatmap (G) and as a volcano plot (H). *n* = 3 biological replicates per condition.

A considerable number of research efforts have attempted to generate retinoid nuclear receptor pathway antagonists, whether through targeting retinoid transport, the atRA synthetic enzymes, or by creating inverse agonists of the RAR/RXR nuclear receptors. These efforts have failed to generate safe therapies that effectively block retinoid signaling *in vivo*.^36^ To validate that the compounds discovered could block ALDH1A3 activity in a relevant disease model, we sought to test a syngeneic tumor model whose *in vivo* tumor growth was shown to be dependent on ALDH1A3 production of atRA and resultant immune suppression.^24^ Thus, mice were injected subcutaneously with T3-MCA sarcoma cells and were randomized by tumor size once tumors became palpable. One group received MBE1.5-formulated diet at 270 ppm (equivalent dose of 40 mg/kg body mass per day based on average food consumption), and the other group received control diet. Mice on MBE1.5-formulated diet showed dramatically reduced tumor growth by day 7 of treatment, and tumors were near static in the treated group through the end of the experiment in contrast to mice on control diet, which exhibited rapid tumor growth and ulceration by the end of the experiment (Figures 4A and 4B). These data correspond well with the growth curves in a previous study.^24^

To determine the breadth of ALDH1A3 activity across human cancers and assess the target engagement of these discovered compounds across tumor models, we profiled a panel of 23 human cancer cell lines with the Aldefluor assay. Data revealed that ALDH1A3 was broadly expressed and that MBE1.5 potently inhibited Aldefluor activity across most solid cancer types except liver cancer (Figure 4C). Next, we assessed whether ALDH1A3 affects cell growth across this broad panel of cancer types by testing proliferation for each cell line with and without MBE1.5. Results from 48-h proliferation assays revealed that there is no detectable effect of ALDH1A3 inhibition on growth or survival across these 23 cell lines (Figure 4D), consistent with our genetic studies using ALDH1A3 knockouts (Figures S1E and S1G).

Given that ALDH1A3 inhibition in ALDH1A3-positive cancer cell lines did not result in any obvious phenotypes *in vitro* despite showing effects *in vivo*, we next sought to perform detailed metabolomic and transcriptomic profiling to assess if either ALDH1A3 inhibition resulted in retinoid-independent effects or if MBE1.5 possessed off-target activity that was not detected by TPP/CETSA. Here, we used ALDH1A3-positive cell lines that were shown to be atRA-insensitive and the ALDH1A3-negative cell line MDA-MB-231 as a control in order to detect retinoid-independent or ALDH1A3-independent effects. MBE1.5 treatment followed by RNA sequencing revealed no detectable changes across the cell lines by multiple pathway analysis tools. Principal component analysis of each sample illustrated that there was little difference in cells treated with MBE1.5 vs. DMSO except that *CYP1* transcripts were commonly upregulated >2-fold in MDA-MB-468 and MDA-MB-231 cells (Figures 4E and 4F). CYP1A1 and CYP1B1 are induced by the aryl hydrocarbon receptor (AhR), which is commonly induced in response to xenobiotic heterocycles in mammary tissue,^37^ and thus AhR induction appears to be the only *bona fide* off-target effect of MBE1.5 treatment of these cell lines. Meanwhile, there appears to be no transcriptomic response to ALDH1A3 inhibition in these retinoid-insensitive cancer cell lines. Metabolomic profiling of polar metabolites in the same cell lines demonstrated that inhibition of ALDH1A3 by MBE1.5 did not directly affect any central metabolic pathways such as glycolysis, tricarboxylic acid cycle, amino acid metabolism, or redox balance (Figures 4G and 4H), thus suggesting that neither MBE1.5 nor ALDH1A3 had detectable retinoid-independent activity in these cell lines outside of AhR activation. Finally, we tested if inhibition of ALDH1A3 resulted in transcriptional feedback of *ALDH1A3* mRNA, thus potentially offering a mechanism of cellular resistance. We treated multiple cell lines with MBE1.5 for 48 h followed by RT-qPCR for *ALDH1A3*. Results showed that ALDH1A3 inhibition had no feedback effect on *ALDH1A3* expression (Figure S5K).

### ALDH1A3 alters CD4 T cell polarization in syngeneic tumors and predicts worse clinical outcomes

**Figure 5:**
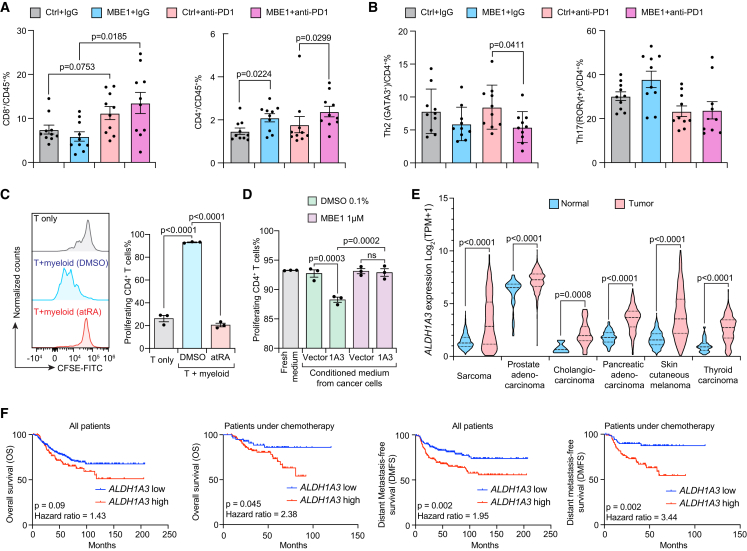
ALDH1A3 suppresses CD4 T cell populations in syngeneic tumors and predicts worse clinical outcomes (A and B) C57BL/6 mice bearing T3-MCA mouse fibrosarcoma tumors were treated with MBE1 or vehicle as well as control IgG or anti-mouse PD-1. Tumors were immunophenotyped for standard T cell subsets (A) and CD4^+^ subsets (B). *n* = 10 biological replicates per arm. Mann-Whitney U test. Data are represented as mean ± SEM. (C) CFSE-stained T cells from C57BL6 mice were cultured alone or with myeloid cells cultured in 0.1% DMSO or 10 nM atRA. Proliferation was quantified by flow cytometry of CFSE. *n* = 3 biological replicates, one-way ANOVA. Data are represented as mean ± SEM. (D) The same CFSE proliferation was conducted on myeloid cells primed by conditioned media from B16F10 cells stably expressing vector or mouse ALDH1A3 that had been treated with 0.1% DMSO or 1 μM MBE1 for 3 days before medium was collected. *n* = 3 biological replicates, one-way ANOVA. Data are represented as mean ± SEM. (E) Analysis of The Cancer Genome Atlas data and GTEX datasets compares *ALDH1A3* expression between tumor samples and normal tissue by RNA sequencing. Student’s *t* test. (F) Kaplan-Meier curves of patients with ER-negative breast cancer stratified by *ALDH1A3* expression into high and low by median within all patients as well as the subgroup that received chemotherapy. Statistics by Cox’s proportional hazards model.

These results suggest that the ALDH1A3 inhibitors from the MBE1 chemotype can effectively block atRA synthesis in tumor cells and thus prevent paracrine secretion of atRA. Here, we aimed to confirm the pharmacodynamic effects of MBE1 *in vivo* using tumor models that were previously validated to depend on ALDH1A3 for suppression of CD4 T cells.^24^ We treated established T3-MCA tumors with vehicle, MBE1 via oral gavage, anti-PD-1 by intraperitoneal injection, or the combination of MBE1 + anti-PD-1. We immunophenotyped the resultant tumors following 7 days of treatment. Data demonstrated that anti-PD-1 elevated CD8 T cell proportions, while ALDH1A3 inhibition increased CD4 T cells as a percentage of total CD45^+^ immune cells (Figure 5A). Within the enriched CD4 T cell population in MBE1-treated groups, the relative proportion of CD4 T cell subpopulations changed, with ALDH1A3 inhibition depleting the Th2 CD4 T cells while it trended toward increasing Th17 CD4 populations (Figure 5B), consistent with previous reports showing that atRA directly suppresses Th17 cells.^38^ Notably, Th1 CD4^+^ T cells were not detectable in this tumor type at the tested time point. Importantly, these findings confirm prior genetic data that suggest ALDH1A3 suppresses anti-tumor CD4 T cells.^24^

To definitely prove that these ALDH1A3 inhibitors block paracrine secretion of atRA to suppress anti-tumor immunity, we established a co-culture model of mouse T cells and mouse myeloid cells that had been treated with tumor cell conditioned media. Assay development showed that mouse T cells were dependent on myeloid cells for proliferation and activity, and atRA addition to those myeloid cells prior to culturing with T cells ablated T cell proliferation (Figure 5C). Next, to simulate paracrine signaling from tumor cells, we collected conditioned media from isogenic B16F10 cells stably expressing either vector or ALDH1A3 and added the media to the myeloid cells prior to culturing with T cells. Whereas vector control media did not affect myeloid cell-stimulated T cell proliferation, conditioned media from ALDH1A3 cells markedly slowed T cell proliferation (Figure 5D), phenocopying the effects of atRA. Importantly, this proliferation block disappeared when conditioned media from MBE1-treated B16F10-ALDH1A3 cells were added to the myeloid culture, indicating that MBE1 treatment blocks ALDH1A3 from producing atRA that diffuses in a paracrine manner to arrest CD4 T cell proliferation (Figure 5D).

We next aimed to identify tumor types that would respond to ALDH1A3 inhibitors in the clinical setting. GTex data revealed that ALDH1A3 expression is low or absent in nearly all normal tissues while it is enriched in discrete tumor types, including sarcoma, prostate cancer, pancreatic cancer, and melanoma (Figure 5E). Expression of ALDH1A3 in breast cancers inversely correlates with the estrogen receptor status,^39^ consistent with our Aldefluor assay showing that expression of ALDH1A3 is elevated in ER^−^ breast cancer cell lines such as HCC-1937, SUM149, SUM159-M1a, and MDA-MB-468 but absent in ER^+^ cell line such as MCF7 (Figures 1A, 1B, and 2A). Stratification within the ER-negative subgroup of patients with breast cancer reveals that ALDH1A3 predicts worse overall survival and distant metastasis-free survival (Figure 5F; Table 3).^40^ This survival data further showed that ALDH1A1 likely participates in the distinct rexinoid pathway, as high expression of ALDH1A1 predicted better outcomes in both ER-positive and ER-negative cancers (Table 3), consistent with decreased tumor growth after ALDH1A1 overexpression in mouse models (Figure 1H).

**Table 3:** High *Aldh1A3* expression is correlated with worse prognosis in ER^−^ breast cancer

Gene	ER^+^		ER^−^		
			All	Chemotherapy	No chemotherapy
*ALDH1A1*	HR	0.67	0.48	2.34	0.48
	*p*	0.00081	0.00039	0.065	0.0071
*ALDH1A3*	HR	0.76	1.85	3.3	1.81
	*p*	0.025	0.004	0.026	0.032

## Discussion

The success of multiple immune checkpoint therapies, such as anti-PD-1 and anti-CTLA4 antibodies, has resulted in unprecedented responses for a minority of patients with cancer. In particular, immune checkpoint therapies have shown a dramatic benefit for patients with metastatic melanomas and lung cancers, yet little benefit in many other prevalent cancers, such as breast or pancreatic cancer.^41^ While current cancer treatments are often effective in treating patients with locally restricted tumors, orthogonal immune-activating therapies are needed to improve outcomes in patients with metastatic or immune-resistant cancers.^42^ Importantly, clinical data from patients with cancer that do not respond to anti-PD-1 therapy show retinol metabolism as the most enriched pathway,^43^ while other studies show that ALDH1A3 is often enriched in patients that do not respond to immune checkpoint inhibitors.^44^ Data presented here demonstrate that ALDH1A3 is expressed and predicts worse outcomes in multiple cancer types that do not respond to immune checkpoint inhibitors, suggesting that therapeutic targeting of ALDH1A3 is a compelling strategy in anti-PD-1-resistant cancers.

Here, we propose that retinoid nuclear receptor signaling is a key central regulatory element of the cancer immune environment that has not been successfully targeted in cancers. Retinoid signaling is validated as a regulator of immune tolerance; a canonical study showed that atRA produced by ALDH1A2 promotes Treg differentiation and Th17 depletion to promote tolerance in the gut.^38^ Accordingly, retinoid agonists are approved for use in various inflammatory skin conditions such as acne or psoriasis as well as autoimmune conditions like discoid lupus.^45^ We show that this mechanism is exploitable in cancer as blockade of retinoic acid production by inhibiting cancer cell-expressed ALDH1A3 allows Th17 T cell expansion while suppressing Th2 T cells. Although both these CD4 subsets show context-dependent roles in tumor immunity, Th2 T cells are broadly acknowledged as part of the immunosuppressive tumor immune environment,^46^^,^^47^ while Th17 cells thought to promote an anti-tumor immune response.^48^

Despite a preponderance of clinical data suggesting that retinoid agonism promotes immune tolerance and accelerates oncogenesis in randomized clinical trials, efforts to therapeutically block retinoid signaling have repeatedly failed.^6^ Prior commercial drug development efforts that focused on the RAR receptors identified three lead compounds with potent preclinical activity (BMS189453, AGN194310, and LY2955303). However, each of these compounds demonstrates unacceptable toxicity, likely due to the requirement that the compounds bind to the challenging hydrophobic pocket of the RAR receptors. Another potential source of toxicity is due to the fact that the RAR receptors modulate non-retinoid functions by binding other nuclear receptors such as PPAR, LXR, or FXR in the apo state.^36^

Another potential conflicting factor that may have prevented the development of retinoid antagonists is the long-standing assumption that the retinoid pathway is tumor suppressive. This hypothesis was derived from the early responses to atRA that were seen in patients with APL before the mechanism of the *RARA-PML* fusion oncogene was understood, but this hypothesis has since been disproven by clinical data from most cancer types.^6^ Interestingly, atRA does slow growth in certain cancer cell lines *in vitro* at supraphysiologic concentrations.^49^ Here, we resolve this paradox by showing that cancers with ALDH1A3 activity concomitantly lose sensitivity to atRA. Thus, ALDH1A3 may be helpful as a predictive marker to both determine which cancers may respond to the inhibitors discovered here and which cancers may be accelerated by retinoid pathway agonists. Interestingly, the paracrine secretion mechanism of ALDH1A3 appears unique to cancer; we have recently demonstrated that ALDH1A3 is responsible for activating cell-autonomous retinoid pathway activation in failing pancreatic β cells.^50^ Developmental biology studies also suggest that ALDH1A3 acts in a cell-intrinsic manner.^9^

In parallel, while ALDH1A3 has long been shown to be associated with worse outcomes in patients with cancer,^12^ its role in retinoid signaling in human tumors has not been conclusively established to date. ALDH1A3 expression is largely absent in syngeneic mouse tumor lines as compared to its broader expression in human cancers. This has necessitated that studies on ALDH1A3 be performed in immune-deficient models where the paracrine effects of tumor-derived atRA would not be identified, thus explaining why a clear mechanism of ALDH1A3 has not yet been identified in cancer.

Previously, drug discovery studies against ALDH enzymes have identified nondrug-like covalent inhibitors, slow turnover substrates that bind the broad ALDH family, and low-potency reversible inhibitors that bind ALDH1A1 but show high cross-reactivity against the broader ALDH1 family.^6^ Key molecules that have been tested *in vitro* include Win18446,^51^ disulfiram,^52^ citral,^8^ DIMATE,^53^ Aldi-1,^54^ CM037 and its derivatives,^55^ NCT-501 and its derivatives,^56^ and the universal negative control compound DEAB. Interestingly, Win18446, DIMATE, and disulfiram have all been tested in humans, but their reactive covalent active moiety results in poor medicinal properties, off-target toxicity (most notably in the liver),^57^ and low potency. Interestingly, studies of disulfiram and DIMATE have reported efficacy across tumor types^58^^,^^59^; however, the poor potency as well as a lack of pharmacodynamic markers leaves the mechanism of these compounds in question. Rather, our data suggest that disulfiram is a potent inhibitor of only ALDH1A1 while DIMATE does not appear to inhibit any of the ALDH1A enzymes at therapeutically relevant concentrations. Only WIN18446 provides a tool compound with which to assess the *in vivo* role of blocking retinoid signaling; however, its lack of enzyme selectivity and known liabilities prevents its repurposing as an immunotherapeutic. Natural products have also been investigated as ALDH1A3 inhibitors, and both citral and modified daidizin analogs show IC_50_ values in the low micromolar range against ALDH1A3; however, *in vivo* studies do not report target engagement with either molecule, and they have known medicinal liabilities.^60^^,^^61^

A series of drug-like, reversible lactone/quinolone structures have been discovered for targeting various ALDH1 isoforms, with the majority of these studies showing IC_50_ values in the 5–20 μM range and limited isoform selectivity.^62^^,^^63^^,^^64^^,^^65^^,^^66^^,^^67^ Only three studies thus far have developed compounds in the 100–500 nM range, and these are more active against ALDH1A1 than other isoforms^68^^,^^69^ with the exception of CM121, a structure with IC_50_ values for ALDH1A2 and ALDH2 in the 500 nM range.^70^ Research in the contraception space has also led to the development of modestly potent ALDH1A1 and ALDH1A2 inhibitors; however, these contain a labile carbonyl with high nonspecific reactivity potential,^70^^,^^71^ further making these undesirable leads. A review of all known inhibitors against the ALDH family exhaustively measured IC_50_ values and confirmed that no known inhibitor exhibited isoform selectivity nor worked in the <200 nM range.^72^ GA11 has more recently been reported with picomolar cytotoxic activity against ALDH1A3 cells and has a reported IC_50_ for ALDH1A3 of 660 nM^28^; however, studies here demonstrate that GA11 has off-target cytotoxic effects and has no ALDH1A3 inhibitory activity up to 10 μM. Finally, additional efforts to modify the CM037 or NCT-501 structures have not yielded drug-like, potent or selective compounds. Notably, a recent patent review highlighted the molecules described here as the only potent and selective inhibitors of ALDH1A3.^27^

In contrast to prior efforts in the ALDH inhibitor field, the series of compounds described here demonstrate excellent potency in the low nanomolar range in cell-based assays, high solubility, low potential to cause drug-drug interactions, are metabolically stable, exhibit low plasma protein binding, high bioavailability, and do not bind across a panel of common off-target proteins. To further validate the compounds described here, we employed a variety of orthogonal techniques to verify that MBE1 is a *bona fide* ALDH1A3 inhibitor, from biophysical, biochemical, cell-based, and pathway-based assays. In summary, there has been no published therapeutic agent outside of the MBE series of compounds described here that can selectively inhibit ALDH1A3.

Expression and prognosis studies have further shown that ALDH1A3 is strongly predictive of poor outcomes across cancer types. Hypermethylation of the ALDH1A3 promoter leading to lower *ALDH1A3* expression was the strongest predictor of favorable outcome in a set of patients with primary glioblastoma.^73^ High ALDH1A3 predicted lymph node metastasis in patients with cholangiocarcinoma.^74^ ALDH1A3 expression is also driven by androgens in prostate cancer, where androgen is the major mitogen for prostate cancer cells.^75^ miR-187 targets ALDH1A3 in prostate cancer, and high miR-187 was correlated with favorable prognosis.^76^ Here, we offer a broad profile of ALDH1A3 activity to show that it is active throughout a variety of solid tumors and that it solely oxidizes retinaldehyde to produce atRA that acts in a paracrine fashion. We further identify the first known ALDH1A3 inhibitor with favorable drug-like traits that can be optimized as a retinoid nuclear pathway inhibitor in clinical trials. Finally, we show that ALDH1A3-produced atRA works in a paracrine immunoregulatory fashion to promote tumor tolerance through depletion of Th17 CD4 T cells and enhancement of Th2 CD4 T cells. Therapies discovered here would enrich CD4 T helper cell activity to promote anti-tumor immune recognition and thus may work either as a single agent or synergistically with CD8 T cell-directed immunotherapies. Future studies will conduct additional profiling to identify the mechanistic effects of atRA on the CD4 T cell population in tumors.

### Limitations of the study

This study focused on the role of ALDH1A enzymes in human cancers, and drug development studies here used human ALDH1A enzymes for key medicinal chemistry optimization. Due to inherent limitations imposed by the human-specific development criteria, the early experiments on ALDH1A3 knockouts in human cancer cell lines were conducted in immunocompromised NSG mice. These models cannot assess the role of ALDH1A3 in a complete immune environment, which is central to the paper’s main hypothesis, and furthermore may not recapitulate potential tumor-intrinsic effects of the retinoid nuclear pathway. Following this limitation, the use of CD34-humanized mice to recapitulate the immune system’s effects suffers from donor-to-donor variability as well as incomplete recapitulation of immune cell subsets, such as monocyte-derived lineages. Further, these systems assume that the retinoid transport and metabolic pathways in the mice are homologous to the human pathway, which has not been robustly demonstrated.

Another key limitation is the quality and levels of vitamin A and downstream retinoid levels in key reagents here, including FBS, mouse diet, and other biological reagents. As retinoids are highly protein bound and are photosensitive, stringent control of retinoid inputs and quality is often used for metabolism studies, with experiments and extractions best performed under gold light conditions. As this is not feasible in the constraints of the equipment and facilities used here, this study may suffer from variability introduced by environmental conditions and inputs.

## Resource availability

### Lead contact

Further information and requests for resources and reagents should be directed to and will be fulfilled by the lead contact, Yibin Kang (ykang@princeton.edu).

### Materials availability

All unique/stable reagents generated in this study are available from the lead contact with a completed materials transfer agreement.

### Data and code availability

• Raw and processed sequencing and proteomics data in this manuscript have been deposited in NCBI GEO (GSE260586) and the PRIDE partner repository (PXD052693).
• No code was generated as part of this study.
• Any additional information required to reanalyze the data reported in this paper is available from the lead contact upon request.

## Acknowledgments

This work was supported by grants from the 10.13039/100001774New Jersey Health Foundation to M.E. and from the 10.13039/100008150Brewster Foundation, the Susan Komen Foundation, the 10.13039/100001006Breast Cancer Research Foundation, the 10.13039/100000048American Cancer Society, and the 10.13039/100009729Ludwig Cancer Research to Y.K. T.R. was funded by 10.13039/100023581National Science Foundation Graduate Research Fellowship Program under grant no. DGE-2039656. We thank Christina DeCoste and the Molecular Biology Flow Cytometry Resource Facility, which is partially supported by the Rutgers Cancer Institute of New Jersey NCI-CCSG P30CA072720-5921.

## Author contributions

M.E. conceived the project, directed studies, and co-wrote the manuscript. M.E., C.F., and Y.W. designed and performed flow cytometry, xenograft, genetic, RT-qPCR, confocal, and bioinformatic experiments and analyzed the data. A.P. and C.B. performed computational docking analysis, X.S. performed metabolomic analysis, and E.D.P. established and tested the MST/DSF assays. M.E. and J.P. supervised medicinal chemistry with X.C. performing compound synthesis and Y.P. performing ADME/PK experiments. M.M. performed TCGA analysis, X.H. and W.W. assisted with experiments, J.E.H. III and C.F. performed TPP experiments, and J.E.H. III and T.R. analyzed TPP data. I.M.C, supervised the TPP experiments. J.Y. and M.K. performed RA quantification. Y.K. conceived and supervised the project, obtained funding, co-wrote the manuscript, and provided experimental advice.

## Declaration of interests

M.E. holds equity and management positions in Kayothera, Inc., a company developing retinoid antagonists. Y.K. holds equity positions and serves as Chair of Scientific Advisory Board in Kayothera, Inc and Firebrand Therapeutics Inc. M.E. and Y.K. hold a patent (US Patent 12,054,475, issued on 08/06/2024) related to the MBE1 series of ALDH1A3 inhibitors.

## STAR★Methods

### Key resources table

**Table:** 

REAGENT or RESOURCE	SOURCE	IDENTIFIER
**Antibodies**		

NPTN	proteintech	Cat# 28022-1-AP; RRID: AB_3086022
ALDH1A3	abcam	Cat# ab129815; RRID: AB_2937054
ALDH1A1	ProteinTech	Cat# 15910-1-AP; RRID: AB_2305276
ALDH1A2	Abcam	Cat# ab75674; RRID: AB_10676130
β-actin	Cell Signaling Technology (Figure 1); Santa Cruz (Figure 2)	CST: Cat# 4967S; RRID: AB_330288 Santa Cruz: Cat# sc-47778; RRID: AB_626632
CD45-PerCP-Cy5.5 (clone 30-F11)	Biolegend	Cat# 45-0451-82; RRID: AB_1107002
CD4-PE (Clone GK1.5)	Biolegend	Cat# 100408; RRID: AB_312693
CD8-FITC (Clone 53–6.7)	Biolegend	Cat# 100705; RRID: AB_312744
CD4-APC-Cy7 (clone GK1.5)	Biolegend	Cat# 100414; RRID: AB_312699
RORγt-PE (clone Q31-378)	BD Biosciences	Cat# 562607; RRID: AB_11153137
GATA3-PE-Cy7 (Clone TWAJ)	eBioscience	Cat# 25-9966-42; RRID: AB_2573568
Tbet-APC (clone 4B10)	Biolegend	Cat# 644814; RRID: AB_10901173
Fc-Block	BD Biosciences	Cat# 553141; RRID: AB_394656
Ly6C-PE (Clone HK1.4)	Biolegend	Cat# 128008; RRID: AB_1186132
IRDye680 secondary antibodies	Licor	Cat# 926-68070; RRID: AB_10956588
IRDye800 secondary antibodies	Licor	Cat# 926-32211; RRID: AB_621843

**Bacterial and virus strains**		

BL21-DE3 E. coli	Thermo Fisher Scientific	Cat# C600003

**Biological samples**		

Human CD34^+^ donor cells	Charles River Laboratories	RRID:SCR_003792

**Chemicals, peptides, and recombinant proteins**		

NAD+	Gold Biotechnology	Cat# *N*-030-1
all- trans retinoic acid (atRA)	Sigma-Aldrich	Cat# R2625
DEAB	Sigma-Aldrich	Cat# D86256
Disulfiram	MedChemExpress (MCE)	Cat# HY-B0240
MCI-INI-3	MedChemExpress (MCE)	Cat# HY-169884
CM010	MedChemExpress (MCE)	Cat# HY-135841
CM037	MedChemExpress (MCE)	Cat# HY-110294
NCT-501	MedChemExpress (MCE)	Cat# HY-18768
GA11	Sigma-Aldrich	Cat# SML2028
DIMATE	This paper	–
Sequencing grade trypsin	Thermo Fisher Scientific	Cat# 90057
Trifluoroacetic acid (TFA)	Thermo Fisher Scientific	Cat# PI28904
Formic acid (FA)	Thermo Fisher Scientific	Cat# 85178
N-dodecyl-β-D-maltoside (DDM)	Thermo Fisher Scientific	Cat# BN2005
Win18446	Tocris Bioscience	Cat# 4736
recombinant human ALDH1A1	This Paper	N/A
recombinant human ALDH1A2	This Paper	N/A
recombinant human ALDH1A3	This Paper	N/A
recombinant mouse ALDH1A3	This Paper	N/A
recombinant human ALDH2	This Paper	N/A
recombinant human ALDH1L1	This Paper	N/A
recombinant human ALDH1L2	This Paper	N/A
recombinant human ALDH3A1	This Paper	N/A
mGM-CSF	PeproTech	Cat# 315-03
mIL-4	PeproTech	Cat# 214-14
D-Luciferin	Gold Bio	Cat# LUCK-100

**Critical commercial assays**		

Aldefluor Assay	StemCell Technologies	01700
EZQuant Reagent	Alstem Bio	CQ01
CellTrace™ CFSE Cell Proliferation Kit, for flow cytometry	Invitrogen	C34554

**Deposited data**		

RNAseq data	NCBI Gene Expression Omnibus (GEO)	Accession number GSE260586
Mass spectrometry proteomics data (TPP)	ProteomeXchange Consortium via the PRIDE partner repository	Dataset identifier PXD052693

**Experimental models: Cell lines**		

A375 (melanoma;female)	ATCC	Cat# CRL-1619; RRID:CVCL_0132
LN229 (glioblastoma;female)	ATCC	Cat# CRL-2611; RRID:CVCL_0393
MDA-MB-468 (breast cancer; female)	ATCC	Cat# HTB-132; RRID:CVCL_0419
NCI-H358 (lung cancer;male)	ATCC	Cat# CRL-5807; RRID:CVCL_1559
SUM159-M1a (breast cancer; female)	Esposito et al.^26^	–
MCF7 (breast cancer; female)	ATCC	Cat# HTB-22; RRID:CVCL_0031
MDA-MB-231 (breast cancer;female)	ATCC	Cat# HTB-26; RRID:CVCL_0062
MDA-MB-231:SCP28 (RARE-Luc retinoid reporter clone)	This paper	N/A
HepG2 cells (Liver cancer; male)	ATCC	Cat# HB-8065; RRID:CVCL_0027
HaCaT (normal human keratinocyte cells;male)	ATCC	RRID:CVCL_0038
HEK293T (embryonic kidney; female)	ATCC	RRID:CVLC_0063
T3-MCA (mouse fibrosarcoma)	Devalaraja et al.^24^	N/A
B16F10 (mouse melanoma)	ATCC	Cat# CRL-6475

**Experimental models: Organisms/strains**		

Mouse: NSG mice	The Jackson Laboratory	NOD.Cg-Prkdcscid Il2rgtm1Wjl/SzJ (RRID:IMSR_JAX:005557)
Mouse: CD34-humanized NCG mice	Charles River Laboratories	NCG-hCD34-donor
Mouse: CD-1 mice	Vital River Laboratories	Strain 201
Mouse: athymic Nu/Nu	Charles River Laboratories	490
Mouse: NOD/SCIDγ	The Jackson Laboratory	005557
Mouse: C57BL/6 Tyr−/−	The Jackson Laboratory	000058

**Oligonucleotides**		

human STRA6 primer (F)	IDT	5′-GGGACAAGTTTCCGGGAGAG
human STRA6 primer (R)	IDT	5′-TCTGGCCCTTCTCCTCCAAT
human ALDH1A1 primer (F)	IDT	5′-TGTTAGCTGATGCCGACTTG
human ALDH1A1 primer (R)	IDT	5′-TTCTTAGCCCGCTCAACACT
human ALDH1A3 primer (F)	IDT	5′-TCTCGACAAAGCCCTGAAGT
human ALDH1A3 primer (R)	IDT	5′-TATTCGGCCAAAGCGTATTC
human GAPDH primer (F)	IDT	5′-GAAGGTGAAGGTCGGAGTC
human GAPDH primer (R)	IDT	5′-GAAGATGGTGATGGGATTTC
Human ALDH1A3 CRISPR Guide RNA 1	IDT	5′-CGTCCCGGAGCAATCTGAAG
Human ALDH1A3 CRISPR Guide RNA 2	IDT	5′-AGCGTCCGCACACACGATGC
Control CRISPR Guide RNA	IDT	5′-ACGGAGGCTAAGCGTCGCAA

**Recombinant DNA**		

Retinoic acid response element-firefly luciferase reporter/GFP (RARE-Luc/GFP)	System Biosciences	TR037PA-P
ALDH1A1 Overexpression plasmid	This paper	–
ALDH1A2 Overexpression plasmid	This paper	–
ALDH1A3 Overexpression plasmid	This paper	–

**Software and algorithms**		

Stata v13	StataCorp LLC	N/A
Microsoft Excel 2017	Microsoft	N/A
Graphpad Prism 8	GraphPad Software LLC	N/A
GLIDE scoring system	Schrödinger	N/A
WinNonLin software	Certara Inc	N/A
FlowJo version X	Becton, Dickinson and Company	RRID:SCR_008520
FACSDiVa version 8.0.1	Becton, Dickinson and Company	RRID:SCR_001456
Python 3.10.8	N/A	N/A
Partek® Flow®	Partek Inc.	N/A

### Experimental model and study participant details

#### Cell lines, cell culture and *in vivo* selection

MDA-MB-231 and its subline SCP28 cells, T3-MCA, 293-T, HaCaT, MDA-MB-468, A375, MCF7, DLD1, HCT116, LN229, HepG2, Huh7, A549, and SW480 cells were cultured in DMEM media supplemented with 10% FBS and pen/strep. DU145 and PC3 cells were cultured in RPMI media supplemented with 10% FBS and pen/strep. The SUM159-M1a cell line,^77^ HCC1937, MDA-MB-453, SUM149, LOVO, and AsPC-1 were grown in Ham’s F12 media supplemented with 10% FBS, 10 μg/mL Insulin and 20 ng/mL EGF as well as pen/strep. No cells lines used here appear in the database of commonly misidentified cell lines (ICLAC). All cell lines were either validated with STR analysis and compared to NCBI repository data where available or were ordered from ATCC and used within 5 passages, except T3-MCA which was a gift from the Haldar Lab (University of Pennsylvania). All cell lines used in the laboratory are confirmed mycoplasma negative by monthly PCR analysis.

#### Cloning, viral production, and transduction

Human and mouse ALDH enzymes were cloned from respective reference RNA, constructed into plasmids using restriction enzyme cloning and confirmed by Sanger Sequencing. Genes were cloned into a vector containing the T7 promoter for bacterial expression and into a lentiviral expression vector for cell line expression as previously described.^78^ Recombinant protein was isolated by inducing BL21-DE3 E. coli (Thermo Fisher) containing the respective plasmid for 20 h at room temperature, shaking, with 300 μM IPTG (Selleck Chem). Cell disruption was achieved via ultrasonication (Φ10, 3s on/6s off, 25min) in 1L of amplified bacterial culture. The target protein was then purified using Ni NTA beads 6FF affinity chromatography. Lysis buffer consisted of 50 mM Na2HPO4, 300 mM NaCl, 1 mM β-ME, pH7.5. The column (2 mL) was washed with 50 mM Na2HPO4, 300 mM NaCl, 1 mM β-ME, 20 mM Imidazole, pH7.5. Elution was performed using 3 mL of 50 mM Na2HPO4, 300 mM NaCl, 1 mM β-ME, 250 mM Imidazole, pH7.5. Following affinity chromatography, the protein underwent further purification via Gel filtration chromatography using a Superdex200 column. Aliquots were quality controlled against a pure internal standard enzyme and/or by western blot to confirm enzyme expression. Purified enzyme was used for biochemical and biophysical assays.

#### Mouse models and xenografts

All procedures involving mice and experimental protocols were approved by the University Institutional Animal Care and Use Committee (IACUC). The study is compliant with all relevant ethical regulations regarding animal research. All xenograft experiments were conducted on 8-12 week-old mice that matched the sex of the corresponding cell line using either athymic Nu/Nu, or NOD/SCIDγ strains. Syngeneic injections were performed on female C57BL/6 Tyr^−/−^ mice. All mice were originally ordered from the Jackson Laboratory and breeding was conducted in an SPF barrier facility except for CD34^+^ humanized mice which were humanized with donor cord blood-derived CD34^+^ HSCs implanted into lethally irradiated NCG mice at Charles River. At 14 weeks post-transplant, mice were monitored for humanization following engraftment and animals utilized here demonstrated >80% humanization as measured by percent of human CD45 from the total CD45^+^ pool. Animals were randomized such that the average humanization was equal. Exact humanization levels were measured at 93.09%, 91.27%, 84.70%, 94.24%, 86.02%, 90.99%, 96.49%, and 89.87% humanization prior to delivery. Detailed information is available as shown below:

**Table:** 

Mouse ID^a^	CRL ID	Humanization ratio^b^	hCD3^+^^c^	hCD4^+^^d^	hCD8^+^^d^	hCD19^+^^e^
01	4B_01-02	93.09%	10.5%	39.00%	55.10%	85.6%
02	4B_01-03	91.27%	4.66%	42.60%	45.30%	91.0%
03	4B_03-03	84.70%	7.47%	53.70%	39.00%	86.8%
04	4B_02-07	94.24%	7.67%	56.10%	36.10%	87.9%
05	4B_02-08	86.02%	8.89%	43.80%	49.30%	86.5%
06	4B_03-02	90.99%	6.59%	46.40%	46.00%	88.70%
07	4B_03-05	96.49%	9.52%	61.70%	31.80%	84.60%
08	4B_05-06	89.87%	20.7%	52.00%	42.70%	71.2%

a Mouse ID: Lab Stamp Identification on tail.

b Percent of human CD45^+^ cells of total lymphocytes.

c Frequency of parent (hCD45^+^).

d Frequency of parent (hCD45^+^CD3^+^).

d Frequency of hCD45^+^ (hCD45^+^hCD3^-^).

No statistical method was used to predetermine the number of animals needed across experiments. Xenograft experiments were conducted using 50k cells suspended in 10 μL PBS for mammary gland injection or 100k cells in 100 μL PBS for subcutaneous injection. Bioluminescent imaging (BLI) and tumor measurement was conducted as previously described.^78^ IV/PO pharmacokinetic analysis of MBE1 and MBE1.5 was conducted on male CD-1 mice from Vital River that had been fasted for 4 h. Sampling was performed from either jugular puncture or tail-vein sampling using BD yellow-capped tubes for serum analysis or K2-EDTA tubes for hematology. Medicated feed was manufactured at Envigo using MBE1.5 powder with a final concentration of 270 ppm. Medicated diet was stored at 4°C for no longer than 6 months at a time.

### Method details

#### Virtual high-throughput screening

To identify potential ALDH1A3 inhibitors with selective inhibition of ALDH1A3 and not ALDH1A1 or ALDH3a1, a virtual high-throughput screening (vHTS) campaign was performed using Aptuit’s virtual compound library. The binding site for ALDH1A3 docking was derived from PDB ID 5FHZ, specifically focusing on the conformation where retinoic acid’s carboxylic group is near the catalytic Cys314, enabling potential covalent inhibitor identification and leveraging key residue differences (Thr315 vs. Ile304 in ALDH1A1 and Val244 in ALDH3A1). Selectivity assessments were also conducted against ALDH1A1 (PDB ID 4X4L) and ALDH3A1 (PDB ID 4L2O). The vHTS employed HYBRID (OEDOCKING 3.2.0.2) for an initial screen of ∼23 million molecules, with top ∼20,000 structures subsequently rescored and refined using MOE (Molecular Operating Environment 2015.10). Docking protocols incorporated specific hydrogen bond mapping to Cys314 and Thr314, and all water molecules were removed from the binding sites. Compounds were then filtered based on drug-like physicochemical properties (MW < 450, cLogP 1–3, H-acceptors 1–5, H-donors 1–3, PSA<140), yielding ∼6,700 unique structures for further MOE docking against ALDH1A1 and ALDH3A1 receptors. Compounds were ranked using a combined score (minimum of HybridRank and MOERank), and a diversity subset of ∼2,000 molecules was selected using Tanimoto similarity (ECFP4 fingerprints, 0.52 cutoff) to avoid over-representation of common motifs like lactams.

#### Aldefluor, biophysical and recombinant activity assay

The Aldefluor assay (StemCell Technologies) was performed according to the manufacturer suggestion with several changes. DEAB (Sigma) was purchased independently, heated to 50°C to liquefy, and then dissolved to 100 mM in ethanol. DEAB was then diluted and used at 1 mM in the assay as the recommended concentration does not inhibit ALDH1A3 activity. In addition, cells were suspended at a concentration of 100,000 cells per mL. Finally, DMSO was added to at 1% final in all cells to improve kinetics of the assay. For compound titrations, concentrated compound stock was added first to tubes and a uniform Aldefluor buffer containing the cell suspension was added to dilute to final volumes. Cells were analyzed by a BD LSRII for flow cytometry and were sorted using a FACSAria Fusion cell sorter (BD Biosciences) equipped with a 70 micron nozzle. Sample and sort collection tubes were kept at 4°C. Gating and analysis were performed with FACSDiVa version 8.0.1 and FlowJo version X software packages using the DEAB condition as the negative control for gating all conditions. DAPI was used as a live-dead indicator.

MicroScale Thermophoresis (MST) assays quantified binding using a Monolith NT.115. ALDH1A3 protein was labeled with NHS-NT647 in 20 mM HEPES pH 8.0, 150 mM KCl, 1 mM TCEP, 0.5 mM NAD+ for 30 min. Binding experiments were conducted in 20 mM HEPES pH 7.5, 150 mM KCl, 0.01% Pluronic F-127, 4 mM GSH, and 2.5% DMSO. Ten titrants were serially diluted (100 μM - 564.5 pM) and measured in duplicate, with data analyzed at a 670 nm/650 nm ratio.

For the recombinant enzyme activity assay, enzyme aliquots were thawed on ice for 4 h prior to analysis and the protocol was performed as described^8^ with the following key changes: 1) Final concentrations of NAD+ (Gold Bio) were 200 μM and BSA were 1 mg/mL 2) Propanaldehyde was used as the substrate for ALDH1A1 and ALDH2. 3) Reaction velocity was monitored through diaphorase (clostridium) coupled reduction of resazurin (100 μM) monitored with ex/em of 560nm/590 nm in order to regenerate NAD+ and thus maintain constant cofactor concentration as well as to avoid high absorbance values of many screening compounds at the NADH absorbance. Assays were conducted at room temperature (controlled to 21-23C) and all buffers were maintained at pH 7.5. For all reactions, pre-incubation time was approximately 2 min while the reaction monitoring was performed over 10 min. Control reagents for the recombinant assay were Win18446 (Tocris), DEAB (Sigma), Disulfiram (MedChemExpress), MCI-INI-3 (MedChemExpress), CM010 (MedChemExpress), CM037 (MedChemExpress), NCT-501 (MedChemExpress), GA11 (Sigma), and DIMATE (see organic synthesis).

#### Thermal proteome profiling (TPP)

##### Sample preparation

Samples were prepared as described previously for thermal proximity coaggregation (TPCA) analysis,^79^^,^^80^ except with minor modifications for CETSA analysis. Treated A375 cells were harvested from 10 × 15 cm plates with treatment with trypsin for 1 min. Cells were washed twice with ice-cold 1x PBS supplemented with 1x HALT protease and phosphatase inhibitor cocktail (Thermo Scientific). Samples were suspended in 600 μL 1x PBS supplemented with 1x HALT protease and phosphatase inhibitor cocktail, and an equal volume (50 μL) of the cell suspension was aliquoted into 10 PCR tubes for thermal denaturation, while the remaining volume was saved as a reference and was not subjected to thermal denaturation. These 10 samples were then subjected to 10-temperature thermal denaturation in 2 PCR Thermal Cyclers (Bio-Rad), where one Thermal Cycler was had rows F, E, D, C, and B set to 36.9°C, 40.2°C, 43.9°C, 46.6°C, and 48.6°C, respectively, while the second Thermal Cycler had rows G, E, D, C, and A set to 52.7°C, 55.3°C, 58.8°C, 61.2°C, and 64°C, respectively. Samples were subjected to these temperatures for 3 min, and then were supplemented with 100 μL 1.5x Kinase buffer (75 mM HEPES, pH 7.5, 15 mM MgCl_2_, 1x HALT, and 3 mM TCEP) and flash frozen in liquid nitrogen until ready for downstream sample preparation. Samples were then thawed on ice, supplemented with 50 μL of 4x IP-DOC lysis buffer (4% TBT, 4% Trition-X100, 1M NaCl, 2% Sodium Deoxycholate, 4x HALT), were transferred to low-bind tubes, and gentle cell lysis was performed by incubating samples on ice for an hour with gentle vortexing every 10 min. Reference samples were lysed in parallel as thermally denatured samples with 5% SDS incubation at room temperature for an hour. All samples were then centrifuged at 20,000xg for 20 min at 4°C. The supernatant for each sample was transferred to a new low-bind tube, reduced with 7.33 μL 500 mM TCEP for 25 min at 56°C, and were then alkylated with 7.33 μL 1M TCEP for 30 min at room temperature in the dark. Samples were then dried down to approximately 100 μL and proteins were precipitated via methanol/chloroform precipitation via the addition of 4x volume HPLC-MS methanol (Fisher Scientific) 1x volume HPLC chloroform (Thermo Scientific), and 3x volume UHPLC-MS water (Thermo Scientific). Mixtures were sonicated for 20 pulses in a cup-horn sonicator, then centrifuged for at max speed for 5 min at 4°C. Supernatants were removed from the protein disks, then washed 2 times with 4x volumes HPLC-MS methanol. Protein pellets were resuspended in 100 μL 100 mM HEPES, pH 8.3, via sonication with 20 pulses. Reference and 36.9°C protein concentration was estimated via the BCA Assay (Thermo Scientific), and enough volume was aliquoted from these samples to achieve 50 μg per sample, and the same volume was removed from the remaining temperatures as was used for the 36.9°C sample per replicate to maintain the proteomic complexity due to thermal denaturation. All samples were adjusted to 100 μL with 100 mM HEPES, pH 8.3, and digested with 1 μg sequencing grade trypsin (Thermo Scientific) at 37°C with shaking at 600 rpm for 16 h. Samples were then dried down to near dryness, suspended in 100 μL 1% sequencing grade trifluoroacetic acid (TFA, Thermo Scientific) made in UHPLC-MS water and desalted via stage-tip desalting.^81^ Briefly, 5 SDB-RPS (Fisher Scientific) disks were cut from using a 16-gauge needle and gently packed into a 200 μL low-bind pipette tip. Tips were conditioned sequentially with 50 μL of 100% LC-MS methanol once, 50 μL 0.1% formic acid (FA, Thermo Scientific) in 19.9% UHPLC-MS water and 80% UHPLC-MS acetonitrile (Buffer 2) once, and 50 μL then 0.1% FA in 99.9% UHPLC-MS water (Buffer 1) twice, centrifuging each wash at 3,000 rpm for 3 min. Insoluble material was precipitated from the samples in 1% TFA by centrifugation at max speed for 5 min. Samples were then loaded onto the condition tips and centrifuged at 3,000 rpm for 5 min. Samples were then sequentially washed with 50 μL of Buffer 1 once, then 50 μL Buffer 2 twice. Samples were eluted with 5% ammonium hydroxide in 15% UHPLC-MS water and 80% UHPLC-MS acetonitrile. Eluted samples were dried down to dryness, resuspended in 50 μL 0.015% N-dodecyl-β-D-maltoside (DDM, Fisher Scientific), 0.1% FA, 4% UHPLC-MS acetonitrile, and 95.885% UHPLC-MS water (resuspension buffer). Peptide concentration for the reference and 36.9°C samples was then estimated via the Scopes method^82^ with a Multiskan SkyHigh plate reader (Thermo Scientific) with a μDrop Duo Plate. For the reference and 36.9°C samples, a sufficient volume to achieve 7.5 μg of peptide equivalent was aliquoted into autosampler vials, and the volume was adjusted to 50 μL to reach 150 ng/μL. The same volume used for the 36.9°C samples was used for the remaining temperatures for each replicate, and the volume was again adjusted to 50 μL to maintain thermal denaturation peptide complexity.

##### LC-MS/MS analysis

LC-MS/MS analysis was performed with a nanoElute 2 attached to a timsTOF Ultra (Bruker) system. The mobile phases were 0.1% LC-MS FA in 99.9% UHPLC-MS water (buffer A) and 0.1% FA in 99.9% UHPLC-MS acetonitrile (Thermo Scientific, buffer B). A one column method with a PepSep ULTRA (25 cm × 75 μm × 1.5 μm) C18 HPLC column (Bruker) and a 10 μm emitter (Bruker) attached to a CaptiveSpray Ultra source was used with a linear 20 min gradient of 3%–34% buffer B at a flow rate of 200 nL/min was for peptide separation. For each sample, 1 μL of sample was injected to achieve 150 ng of peptide material injected on column. For data independent acquisition parallel accumulation serial fragmentation (dia-PASEF), the MS1 settings were set to start at 100 *m/z* and end at 1,700 *m/z* in positive ion polarity. For the TIMS settings, the mode was set to custom, with a starting 1/K_0_ of 0.65 V s/cm^2^ and an ending 1/K_0_ of 1.46 V s/cm^2^. The ramp time was set to 50 ms with a 100% duty cycle and a ramp rate of 17.80 Hz. An optimized 3 × 16 dia-PASEF was generated from previous HeLa standard (Thermo Scientific) injections and is shown in Table S1.

##### Protein assembly from DIA data

Data-Independent Acquisition (DIA) data was analyzed with DIA-NN,^83^ where a spectral library search was performed with an *in silico* spectral library that was generated via a modified AlphaPeptDeep library generation tool^84^ using a *Homo sapiens* FASTA database (downloaded 02/2024) and an in-house generated contaminant database. The fragment ion *m/z* range was set to 200-1,800, variable PTMs included were N-terminal methionine excision and N-terminal acetylation, *in silico* digestion with trypsin with cleavage at lysine and arginine for the FASTA database, a maximum of 1 missed cleavages, a peptide length between 7 and 30 amino acids, a precursor *m/z* range of 300 to 1,800, precursor charge state of 1–4, a fixed cysteine carbamidomethylation, a fixed mass accuracy of 15 ppm for MS2 and 20 ppm for MS1 scans, and a spectral library was generated from the DIA runs. Relevant peptides and unnormalized proteins were shown in Tables S2 and S3, respectively.

#### Analysis of TPP data

##### Data processing

To process the DIA-NN searched CETSA data first all proteins with less than two identified peptides were discarded. Next, known contaminates were removed from the dataset. To normalize this processed CETSA data, scaling factors for each temperature for each run (a single temperature of a single condition and replicate) were calculated. First, the mean precursor quantity (from the Precursors. Identified column of the ‘.stats’ DIA-NN output file) for all temperatures and replicates within a single condition was calculated. The precursor quantity of each run within the given condition was then divided by this mean precursor quantity, generating scaling factors. All protein abundances within a given run were then multiplied by that run’s scaling factor. Next, for each individual protein, the protein abundances for each temperature and replicate within a given condition was divided by the median abundance the given protein, calculated for each individual condition. Next, a scaling factor was calculated using the reference samples. The median abundance of each protein within the replicate reference samples for a given condition was calculated. Each protein within that given set of replicates for that given condition was then divided by its respective median reference value. Proteins for which no reference-based median abundance could be calculated were instead divided by the median of all protein median reference abundance. Finally, proteins within a given replicate and condition which had more than three missing values across all ten temperatures were discarded, unless they had values for all of the first five temperatures. CETSA data processing and analyses were performed using Python 3.10.8, utilizing the Python libraries Numpy, Scipy, Pandas, Seaborn, and Matplotlib.

##### Calculating Protein T_m_ and ΔT_m_

To calculate a protein’s melting temperature (T_m_), protein melting curves were first assessed to determine if there was a temperature at which a protein’s relative abundance was 0.5 ± 0.05. If such a temperature was identified, then that temperature was used as the protein’s T_m_, otherwise, spline-based curve fitting was performed. Using interpolation, the melting temperature was calculated. If interpolation could not identify a melting temperature, the protein’s T_m_ was set to 64°C, the maximum temperature used in the CETSA experiment. Melting shifts (ΔT_m_) were calculated by taking the difference in a protein’s calculated T_m_ between two conditions. Normalized abundances of soluble proteins at every temperature were shown in Table S4. Processed T_m_ values as well as statistical tests were listed in Table S5 (DMSO vs. 100 nM MBE1) and Table S6 (DMSO vs. 1 μM MBE1).

#### Cellular thermal shift assays (CETSA)

CETSA was performed according to protocols described before.^85^ Briefly, 6 × 10cm dishes of A375 cells were collected and treated with 100 nM MBE1 or 0.1% DMSO for 1h at 37°C. Afterward, cells were pelleted through centrifugation at 300*g* for 5 min. Cells were then washed with ice-cold PBS and resuspended in 800 μL of PBS. Cell suspension was aliquoted to 7xPCR tubes with 100 μL each and subjected to thermal denaturation at 46.3, 48.6, 52.2, 56.5, 60.1, 62.5, 64.0°C for 3 min. The resulting sample was snap frozen in liquid nitrogen followed by incubation at room temperature until fully thawed. This procedure was performed twice followed by brief vortexing and mixing to release soluble proteins from the cells. Samples were then transferred to 1.5 mL Eppendorf tubes and centrifuged at 20,000g for 20 min at 4°C, 90 μL of the resulting supernatant was collected into another new 1.5 mL Eppendorf tube and subjected to protein analysis by western blot (WB). NPTN (proteintech #28022-1-AP) and ALDH1A3 (abcam #ab129815) were used to detect target proteins while GelCode Blue Stain Reagent (Thermofisher scientific #24590) was used to stain for total proteins loaded in the WB analysis.

#### Metabolomics

Cells were grown to 90% confluence in 6 cm tissue culture dishes over 24 h, at which point cells were washed twice with warmed PBS and then plated with fresh media. Cells were treated with MBE1.5 or DMSO concurrently. Media was removed after 3 h, and metabolites were extracted by adding a solution of 1 mL of ice-cold 40:40:20 (Acetonitrile:Methanol:Water, All HPLC grade) + 0.5% Formic acid solution (v/v). Plates were rocked at 4°C for 20 min, followed by addition of 50 μL of 15% (m/v) Ammonium Bicarbonate. Supernatants were scraped and spun at 15000 x g for 10 min followed by LC-MS analysis as previously published.^86^ Processed data is provided in Table S7. All-*trans* retinoic acid was quantified as previously described.^87^

#### qPCR, western blot, and proliferation assay

Quantitative real-time PCR (qRT-PCR) was performed using the SYBR green mastermix protocol on cDNA generated using the SuperScript IV cDNA assembly kit (Invitrogen) and the primers for human *STRA6* (F 5′-GGGACAAGTTTCCGGGAGAG, R 5′-TCTGGCCCTTCTCCTCCAAT), human *ALDH1A1* (F 5′-TGTTAGCTGATGCCGACTTG, R 5′-TTCTTAGCCCGCTCAACACT), human *ALDH1A3* (F 5′-TCTCGACAAAGCCCTGAAGT, R 5′-TATTCGGCCAAAGCGTATTC), and human *GAPDH* (F 5′-GAAGGTGAAGGTCGGAGTC, R 5′-GAAGATGGTGATGGGATTTC). RNA was purified using the respective Qiagen kits. RNA counts were normalized to GAPDH mRNA and then relative cell line values were compared to values from a universal human reference (Agilent 740000). western blotting was performed using standard reducing conditions followed by transfer to PVDF membranes. Membranes were blocked in 5% milk in TBS-T, probed with antibodies for ALDH1A1 (ProteinTech), ALDH1A2 (Abcam), ALDH1A3 (Abcam) or β-actin (Santa Cruz), and imaged on the Licor Odyssey CLX system using Licor-supplied IRDye680 and IRDye800 secondary antibodies. Uncropped, raw western blot images are shown in Figure S7. Cell proliferation rates were quantified using the EZQuant reagent (Alstem Bio).

#### RNA sequencing

Cells were grown in 10 cm plates to 50% confluence and media was changed one day before analysis. At 6 h prior to RNA extraction, MBE1.5 or DMSO was added and rocked. RNA was extracted using the Qiagen RNAeasy kit after 9 h of incubation.

RNAseq results were analyzed by Partek Flow. Raw reads were aligned using STAR 2.7.8a, followed by quantification to annotation model hg38 – Ensembl Transcripts release 110. After noise filtering, counts were normalized by FPKM method and added 1.0. Principal Component Analysis (PCA) analysis was performed with equal contributions from features, and hierarchical clustering was done based on Euclidean distance metric.

#### Tumor infiltrating immune cell profiling

Tumors were dissected and dissociated using the tumor dissociation kit (Miltenyi Biotec #130-096-730) followed by mechanical dissociation by gentleMACS Octo Dissociator with Heaters. After filtration through 70 μm strainer, cells were subject to red blood cell lysis in RBC lysis buffer (Biolegend 420302), followed by another round of cell filtration through 70 μm strainer to get single cell suspension. Cells were then resuspended in 1 μg/ml Fc-Block (BD 553141) containing PBS and incubated at room temperature for 10 min. Antibody mix used at 1:200 is then added to the cell suspension. After 10–20 min incubation at room temperature, cells were washed and resuspended in PBS. Cell samples were then loaded through Cytkick autosampler and data was acquired on Attune NxT flow cytometer. All flow cytometry data was analyzed by Flowjo.

Reagents for flow cytometry staining used in this study are: CD45-PerCP-Cy5.5 (clone 30-F11, #45-0451-82), CD4-PE (Clone GK1.5 100408), CD8-FITC (Clone 53–6.7, 2002714), CD4-APC-Cy7 (clone GK1.5, 100414), RORγt-PE(clone Q31-378, 562607), GATA3-PE-Cy7 (Clone TWAJ, 25-9966-42), Tbet-APC(clone 4B10, 644814), Fixable Viability Dye eFluor 506 (65-0866-14).

#### T cell proliferation assays

Bone marrow derived monocytes were enriched using MACS isolation (#130-100-629) and >95% purity of monocytes was confirmed by staining with Ly6C-PE (Clone# HK1.4; Biolegend 128008). Monocytes were cultured in RPMI1640 at 1M/well in a 6-well plate. 20 ng/ml mGM-CSF (peprotech) and 20 ng/ml mIL-4 (peprotech) were used to differentiate monocytes to monocyte derived dendritic cells (MoDCs). 100 nM atRA or 0.1% DMSO was used to treat the cells. 3 days later, floating cells were collected and 20,000 of them were cocultured with 100,000 CD4^+^ T cell labeled with CFSE (Thermo fisher scientific) at 1:5000. T cell proliferation was examined ∼72hrs after the coculture. When conditioned medium was used in differentiating MoDCs, conditioned medium was first collected from 500,000 B16-vector control cells vs. B16-mALDH1A3 cells with or without 1 μM MBE1 treatment cultured in 1.5 mL medium in a 6-well plate for ∼24hrs. Fresh RPMI1640 and conditioned medium were mixed 1:1 before applying to MoDC culture. In parallel, fresh RPMI1640 medium alone is used as a control. MoDCs were collected 3 days later and subjected to coculture experiments following the procedures mentioned above.

#### Organic chemistry

MBE1 is synthesized following the procedures shown in Figure S8A, with NMR validation report shown in Figure S8B.

Step 1: Stage 1: To a stirred solution of NaNO2 (7.41 g, 107.47 mmol, 2 eq) in water H_2_O (80 mL) was added Amberlyst A26-OH (28 g). The resulting mixture was stirred at 25°C for 0.5 h, and then polymer-supported resin was filtered and washed with water until the pH of filtrate became neutral. The polymer-supported nitrite was got. Stage 2: To a solution of 4-fluoro-1,2-dinitro-benzene (10 g, 53.74 mmol, 1 eq) and methyl prop-2-enoate (4.63 g, 53.74 mmol, 4.84 mL, 1 eq) in MeOH (100 mL) was added *p*-toluenesulfonic acid monohydrate (10.22 g, 53.74 mmol, 1 eq), Pd(OAc)2 (193.02 mg, 859.77 μmol, 0.016 eq) and was slowly added polymer-supported nitrite. The mixture was stirred at 60°C for 1 h. TLC indicated 4-fluoro-1,2-dinitro-benzene was consumed completely and one new spot formed. The reaction mixture was filtered and filtrate concentrated under reduced pressure to give a residue. The crude product methyl (E)-3-(4-fluoro-2-nitro-phenyl)prop-2-enoate (6 g, crude) was obtained as a yellow solid.

Step 2: To a solution of methyl (E)-3-(4-fluoro-2-nitro-phenyl)prop-2-enoate (6 g, 26.65 mmol, 1 eq) in MeOH (50 mL) was added Pd/C (800 mg, 26.65 mmol, 10% purity, 1.00 eq) under H_2_ atmosphere. The suspension was degassed and purged with H2 for 3 times. The mixture was stirred under H2 (15 Psi) at 40°C for 1 h. TLC indicated methyl (E)-3-(4-fluoro-2-nitro-phenyl)prop-2-enoate was consumed completely and one new spot formed. The reaction mixture was filtered and filtrate concentrated under reduced pressure to give a residue. The crude product was triturated with PE:EA = 50:1 (6 mL) at 25°C for 10 min. Compound 7-fluoro-3,4-dihydro-1H-quinolin-2-one (3 g, 18.16 mmol, 68.17% yield) was obtained as a white solid.

Step 3: To a solution of 7-fluoro-3,4-dihydro-1H-quinolin-2-one (3 g, 18.16 mmol, 1 eq) in H_2_SO_4_ (20 mL) was added KNO_3_ (1.84 g, 18.16 mmol, 1 eq) at 0°C. The mixture was stirred at 25°C for 1 h. TLC indicated 7-fluoro-3,4-dihydro-1H-quinolin-2-one was consumed completely and one new spot formed. The reaction mixture was cooled at 0°C and the resulting solution was stirred for 15 min at 0°C. The reaction was quenched by adding 100 mL of H_2_O/ice. The mixture was filtered and filter cake was concentrated under reduced pressure to give a residue. The crude product 7-fluoro-6-nitro-3,4-dihydro-1H-quinolin-2-one (2.5 g, 11.90 mmol, 65.49% yield) was obtained as a white solid

Step 4: To a solution of 7-fluoro-6-nitro-3,4-dihydro-1H-quinolin-2-one (1.5 g, 7.14 mmol, 1 eq) in MeOH (10 mL) was added Pd/C (200 mg, 7.14 mmol, 10% purity) under H2 atmosphere. The suspension was degassed and purged with H2 for 3 times. The mixture was stirred under H2 (15 Psi) at 25°C for 1 h. TLC indicated 7-fluoro-6-nitro-3,4-dihydro-1H-quinolin-2-one was consumed completely and one new spot formed. The reaction mixture was filtered and filtrate was concentrated under reduced pressure to give a residue. The crude product was triturated with PE:EA = 10:1(11 mL) at 25°C for 10 min. Compound 6-amino-7-fluoro-3,4-dihydro-1H-quinolin-2-one (0.8 g, 4.44 mmol, 62.21% yield) was obtained as a white solid.

Step 5: To a solution of 6-amino-7-fluoro-3,4-dihydro-1H-quinolin-2-one (100 mg, 555.00 μmol, 1 eq) and 3-ethylpyridine-4-carboxylic acid (83.90 mg, 555.00 μmol, 1 eq) in DMF (5 mL) was added EDCI (127.67 mg, 666.01 μmol, 1.2 eq) and Py. (65.85 mg, 832.51 μmol, 67.20 μL, 1.5 eq). The mixture was stirred at 25 °C for 12 h. LC-MS showed 6-amino-7-fluoro-3,4-dihydro-1H-quinolin-2-one was consumed completely and the desired MS was detected. The reaction mixture was diluted with H_2_O (10 mL) and extracted with EtOAc 15 mL (5 mL ∗ 3). The combined organic layers were washed with brine 10 mL, dried over [Na2SO4], filtered and concentrated under reduced pressure to give a residue. The residue was purified by prep-TLC (SiO2, PE: EA = 0:1). Compound MBE1 (3-ethyl-N-(7-fluoro-2-oxo-3,4-dihydro-1H-quinolin-6-yl)pyridine-4-carboxamide; 65 mg, 203.72 μmol, 36.71% yield, 98.2% purity) was obtained as a white solid.

MBE1.5 is synthesized following the procedures shown in Figure S8C, with NMR validation report shown in Figure S8D.

To a solution of 6-amino-7-fluoro-3,4-dihydro-1H-quinolin-2-one (119.98 mg, 665.89 μmol, 1 eq) and 2-ethylbenzoic acid (100 mg, 665.89 μmol, 1 eq) in DMF (5 mL) was added EDCI (153.18 mg, 799.07 μmol, 1.2 eq) and Py. (79.01 mg, 998.84 μmol, 80.62 μL, 1.5 eq) at 0°C. The mixture was stirred at 25°C for 2 h. LC-MS showed 6-amino-7-fluoro-3,4-dihydro-1H-quinolin-2-one was consumed completely and the desired MS was detected. TLC indicated two new spots were detected. The reaction mixture was diluted with H_2_O (10 mL) and extracted with EtOAc 15 mL (5 mL ∗ 3). The combined organic layers were washed with brine 10 mL, dried over [Na_2_SO_4_], filtered and concentrated under reduced pressure to give a residue. The residue was purified by prep-TLC (SiO_2_, PE: EA = 0:1). Compound MBE1.5 (2-ethyl-N-(7-fluoro-2-oxo-3,4-dihydro-1H-quinolin-6-yl)benzamide; 60 mg, 182.80 μmol, 27.45% yield, 95.16% purity) was obtained as a white solid.

DIMATE is synthesized following the procedures shown in Figure S8E, with NMR validation report shown in Figure S8F.

Step 1: The mixture of 3-chloro-3-methyl-but-1-yne (2 g, 19.50 mmol, 2.19 mL, 1 eq) and N-methyl methanamine (7.91 g, 70.20 mmol, 8.89 mL, 40% purity, 3.6 eq) was stirred at 20 °C for 12 h. TLC indicated Starting material was consumed completely and one new spot formed. The reaction mixture was filtered and the filter cake was concentrated under reduced pressure to give a residue. Compound N, N,2-trimethylbut-3-yn-2-amine (0.5 g, 4.50 mmol, 23.06% yield) was obtained as a yellow solid.

Step 2: To a solution of N, N,2-trimethylbut-3-yn-2-amine (0.1 g, 899.41 μmol, 1 eq) in THF (20 mL) was added n-BuLi (2.5 M, 2.52 mL, 7 eq) at −70 °C for 1 h. CO2 (1 g, 22.72 mmol, 25.26 eq) was bubbled into the mixture at 0°C for 15 min. The mixture was stirred at 0 °C for 1 h. The reaction mixture was used in the next step without further purification. The mixture [4-(dimethylamino)-4-methyl-pent-2-ynoyl] oxylithium (23 mL solution) was obtained.

Step 3: To a solution of [4-(dimethylamino)-4-methyl-pent-2-ynoyl] oxylithium (806.82 μmol, 23 mL, 1 eq) in THF (10 mL) was added isobutyl carbonochloridate (550.96 mg, 4.03 mmol, 527.74 μL, 5 eq). The mixture was stirred at 0 °C for 1 h. LC-MS showed [4-(dimethylamino)-4-methyl-pent-2-ynoyl] oxylithium was consumed completely and desired mass was detected. The reaction mixture was used in the next step without further purification. The mixture isobutoxycarbonyl 4-(dimethylamino)-4-methyl-pent-2-ynoate (30 mL solution) was obtained.

Step 4: To a solution of isobutoxycarbonyl 4-(dimethylamino)-4-methyl-pent-2-ynoate (705.03 μmol, 30 mL, 1 eq) in THF (10 mL) was added NaSMe (285 mg, 4.07 mmol, 259.09 μL, 5.77 eq). The mixture was stirred at 20 °C for 2 h. TLC indicated isobutoxycarbonyl 4-(dimethylamino)-4-methyl-pent-2-ynoate was consumed completely and many new spots formed. The reaction mixture was diluted with H2O 20 mL and extracted with EtOAc 20 mL (10 mL ∗ 2). The combined organic layers were washed with brine 20 mL (10 mL ∗ 2), dried over Na2SO4, filtered and the filtrate concentrated under reduced pressure to give a residue. The residue was purified by prep-TLC (SiO2, Petroleum ether/Ethyl acetate = 1:1). Compound S-methyl 4-(dimethylamino)-4-methyl-pent-2-ynethioate (20 mg, 107.94 μmol, 15.31% yield) was obtained as a yellow oil.

Step 5: To a solution of S-methyl 4-(dimethylamino)-4-methyl-pent-2-ynethioate (20 mg, 107.94 μmol, 1 eq) in HCl/EtOAc (1 mL). The mixture was stirred at 20 °C for 5 min. LCMS indicated desired compound. The reaction mixture was concentrated under reduced pressure to give a residue. Compound S-methyl 4-(dimethylamino)-4-methyl-pent-2-ynethioate (20 mg, 90.19 μmol, 83.56% yield, HCl) was obtained as a white solid. GC MS, 100% purity, no signal by LCMS. 1H NMR (400 MHz, METHANOL-d4) δ ppm 3 (s, 6H), 2.46 (s, 3H), 1.78 (s, 6H).

### Quantification and statistical analysis

All statistical comparisons were conducted with Stata v13 (Mann-Whitney U, repeated measures ANOVA, regression, and Cox’s Proportional Hazards), Microsoft Excel 2017 (Student’s T-test), or Graphpad Prism 8. Results are reported as mean ± standard error of the mean (S.E.M.) for bar graphs or line graphs with accompanying individual data points for *n* < 6. For all animal experiments, animals were only excluded if they died or had to be sacrificed according to the pre-defined criteria listed in the IACUC protocol. Two-sided, unpaired Student’s t test was used for all qPCR, flow cytometry data, and other normal data. One-way ANOVA is used when there are three or more groups in an experiment and *p*-values between groups are calculated using the multiple comparison feature in Graphpad Prism 8. Survival data for patient datasets were analyzed by Cox’s proportional Hazards model to generate hazard ratios and their associated *p*-values. When asterisks are used to represent statistical significance, ∗*p* < 0.05, ∗∗*p* < 0.01, ∗∗∗*p* < 0.005.

## Supplemental information

Document S1. Figures S1–S8

Table S1. Data-independent acquisition windows set on the TimsTOF Ultra instrument

Table S2. Peptides identified by LC-MS/MS

Table S3. Unnormalized proteins identified by LC-MS/MS

Table S4. Normalized protein abundances at different temperatures

Table S5. Tm shift and statistical significance of DMSO vs. MBE 100 nM

Table S6. Tm shift and statistical significance of DMSO vs. MBE 1 μM

Table S7. Raw counts of each listed metabolite across cell lines treated with MBE1 or DMSO
