# Supplementary material for: Development of retinoid nuclear receptor pathway antagonists through targeting aldehyde dehydrogenase 1A3
Source: iScience. 2025 Oct 3;28(11):113675. doi: 10.1016/j.isci.2025.113675 (PMC12595434; doi:10.1016/j.isci.2025.113675)
Supplement: Document S1. Figures S1–S8 [file mmc1.pdf]

iScience, Volume *28*

Supplemental information

Development of retinoid nuclear receptor pathway antagonists through targeting aldehyde dehydrogenase 1A3

Mark Esposito, Cao Fang, Yong Wei, Alfonso Pozzan, Claudia Beato, Xiaoyang Su, Josiah E. Hutton III, Tavis Reed, Xiang Hang, Enrico D. Perini, Wen Wang, Xiaobing Cheng, Yan Pan, Jianshi Yu, Maureen Kane, Malini Manoharan, John Proudfoot, Ileana M. Cristea, and Yibin Kang

Supplemental Figures

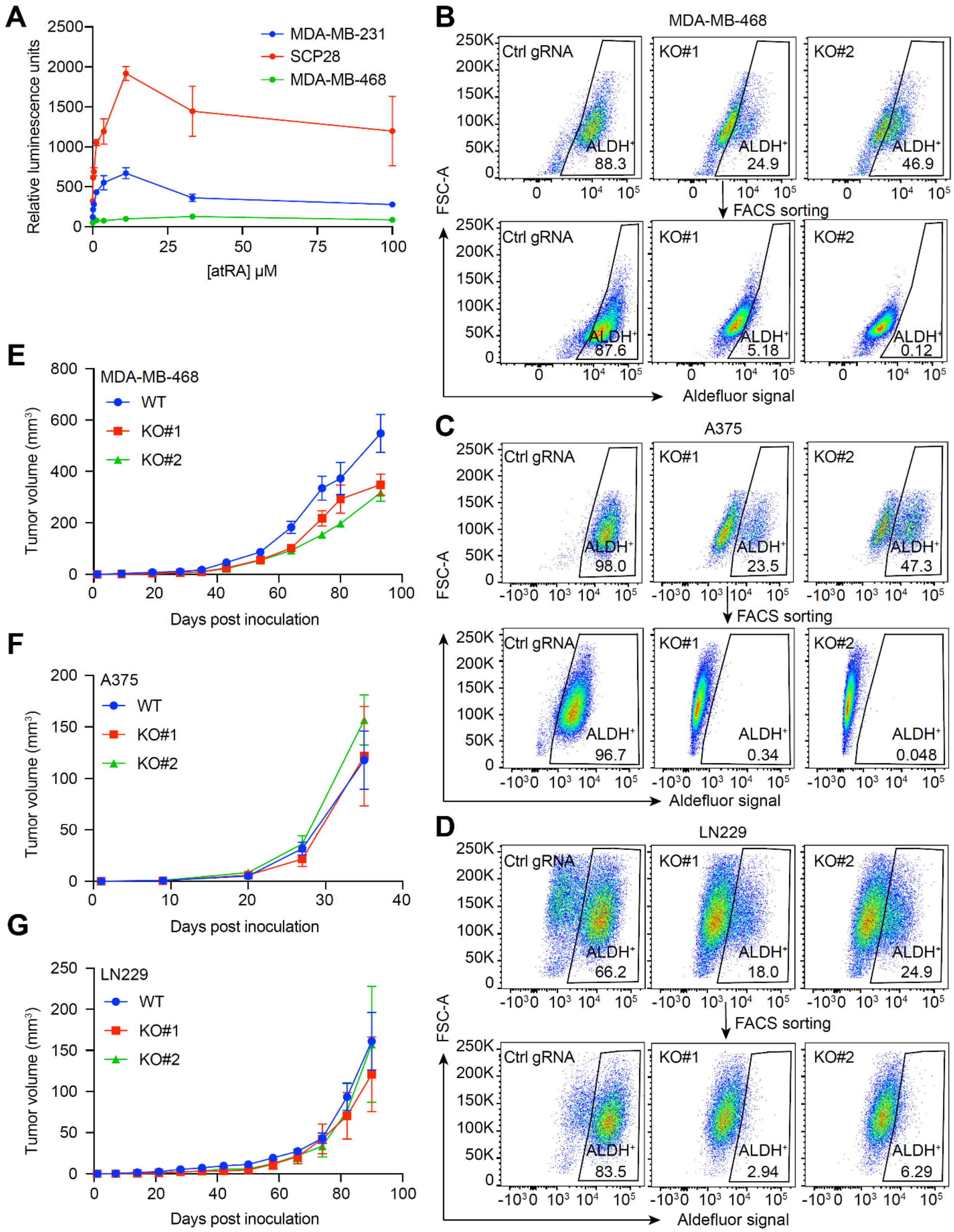

Figure S1. ALDH1A3 is expressed by tumor cells to generate retinoic acid. A, Retinoic acid response reporter driving firefly luciferase expression was stably introduced and measured by luminescence in MDA-MB-468, MDA-MB-231 and its subline SCP28 following a 24 hour dose-titration with atRA. n = 4 biological replicates. Data are represented as mean ±SEM**. B-D**, Cancer cell lines were stably transduced with CRISPR-Cas9 lentivirus using two separate guide sequences targeting *ALDH1A3* or a scrambled control guide RNA. MDA-MB-468 (B), A375 (C), and LN229 (D) cells were then quantified for Aldefluor activity following selection and stable culture to passage 5 (top panels of each). Cells were expanded, and FACS was used to sort Aldefluor populations based either on the negative population for ALDH1A3 knockouts or the total population for the scramble guides. At passage 3 post-sorting, each population was then assessed for Aldefluor activity (bottom panels)**. E-G**, Stably-selected and sorted cells from (B-D) were implanted into mice either orthotopically via mammary fat pad injection into female NSG mice (MDA-MD-468, E) or subcutaneously implanted into NSG mice (A375, F. LN229, G.). Mice were followed for tumors by weekly tumor measurement (n = 8 mice per group (E), n = 8 mice per group in (F), and n = 9 mice/group in (G). Data are represented as mean ±SEM.

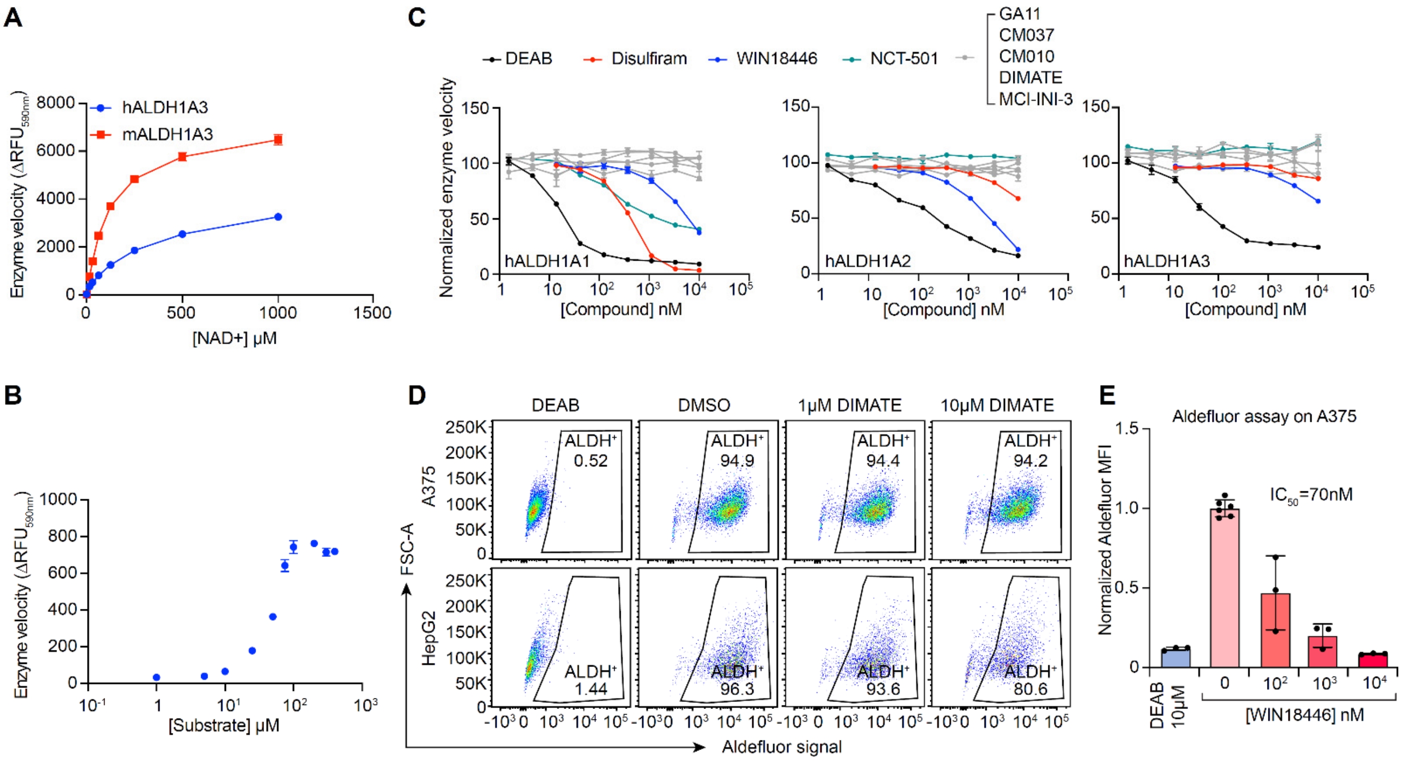

Figure S2. Kinetic profiling of ALDH1A enzymes across cofactors, substrates and known inhibitors. A, Enzyme kinetics of human and mouse ALDH1A3 across varying concentration of NAD+ cofactor. n = 3 biological replicates. Data are represented as mean ±SEM**. B**, Michaelis-Menten kinetic calculation of aldehyde substrate K_m for human ALDH1A3 with NAD^+ at 200 μM. n = 3 biological replicates. Data are represented as mean ±SEM. **C**, Dose-titration curves of each ALDH1A isoform in the presence of the indicated inhibitors. DEAB, Disulfiram, WIN18446 and NCT-501 showed measurable inhibition and are highlighted in color. GA11, CM037, CM010, DiMATE, MCI-INI-3 are all shown with gray color without distinction due to a lack of inhibitory activity. n=3 biological replicates**.** Data are represented as mean ±SEM. **D**, Aldefluor profiling of A375 ($ALDH1A3^+$) and HepG2 ($ALDH1A1^+$) cells in the presence of 1 mM DEAB, 0.1% DMSO, or DIMATE at 10μM or 100nM. **E**, IC50 calculation for WIN18446 using Aldefluor profiling of A375 cells. DEAB graphed as negative control. n=3 biological replicates. Data are represented as mean ±SEM.

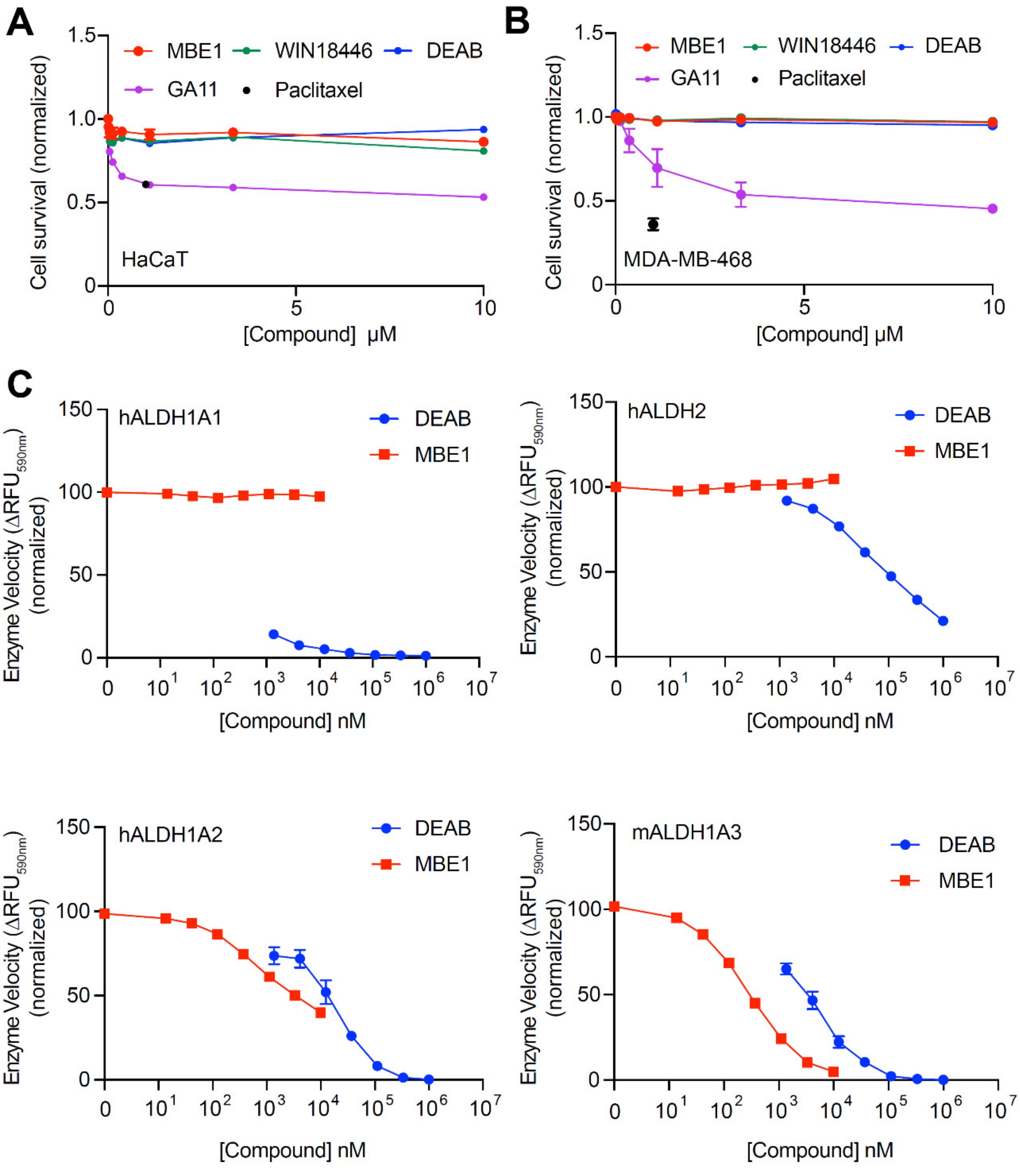

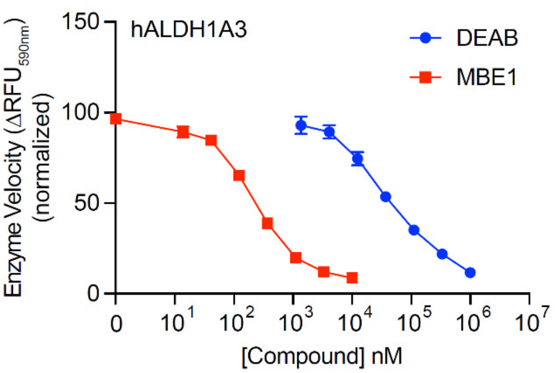

Enzyme	DEAB IC_{50} (nM)	MBE1 IC_{50} (nM)
hALDH1A1	65.8	No activity
hALDH1A2	18,887	845
hALDH1A3	37,201	219
mALDH1A3	3053	283
hALDH2	77049	No activity

Figure S3. MBE1 is a non-cytotoxic, specific inhibitor of ALDH1A3. A-B, Dose-titration cytotoxicity analysis of compounds MBE1, Win18446, GA11, and DEAB was performed on HaCaT (A) and MDA-MB-468 (B) cells over a 24 hour incubation. Paclitaxel at 1 μM was used as a positive control graphed at a single point. n=3 biological replicates. Data are represented as mean ±SEM**. C**, Specificity analysis of MBE1 against human ALDH1A1, ALDH1A2, ALDH1A3, ALDH2 and mouse ALDH1A3 in the biochemical kinetic assay. IC_{50} by 4 parameter calculation included as a summary table. n = 3 biological replicates. Data are represented as mean ±SEM.

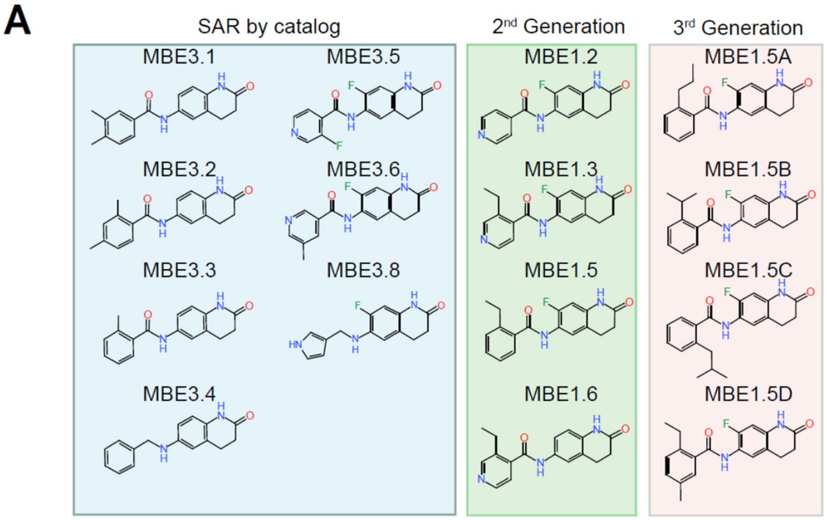

B

Compound	IC_{50} hALDH1a3	IC_{50} mALDH1a3
MBE1	144	244
MBE2	2197	1168
MBE3.1	>10000	>10000
MBE3.2	>10000	>10000
MBE3.3	5000	2949
MBE3.4	>10000	>10000
MBE3.5	>10000	5061
MBE3.6	>10000	>10000
MBE3.8	>10000	>10000
MBE1.2	>10000	>10000
MBE1.3	1500	4602
MBE1.5	83	149
MBE1.6	95	266
MBE1.5A	64	351
MBE1.5B	2712	1854
MBE1.5C	36	322
MBE1.5D	54	154

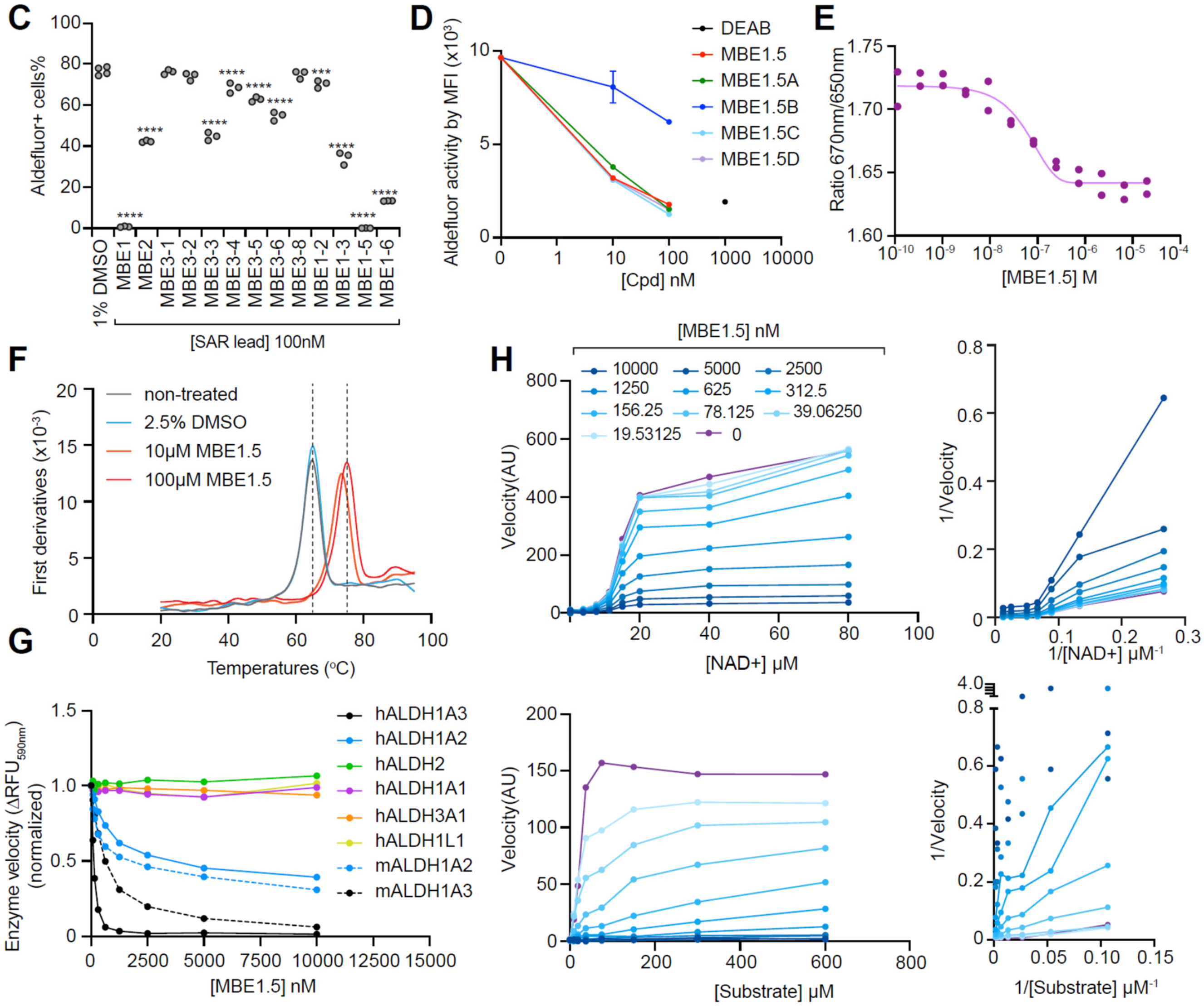

Figure S4. Medicinal chemistry approach identifies structure-activity relationship for ALDH1A3 inhibitors. A, B, Highly similar structures to MBE1 were either ordered from known chemical vendors (1st generation) or synthesized *de novo* (2nd and 3rd generation) to develop an ALDH1A3 structure-activity relationship. IC_{50} values for each compound in the kinetic assay are listed (B)**. C**, Aldefluor profiling of compounds from (A) at 100 nM in A375 cells identifies cellular activity using DEAB as negative and DMSO as positive controls. n = 3 biological replicates**.** Statistics by Student's T-test. ****p<0.001. Data are represented as mean ±SEM. **D**, A dose titration on MBE1.5 and its derivatives in the Aldefluor assay using SUM159-M1a-ALDH1A3 cells, with DEAB as negative and DMSO as positive controls. n = 3 biological replicates. Data are represented as mean ±SEM**. E**, K_d of MBE1.5 to ALDH1A3 was calculated by the MST assay to assess native dissociation constant for binding**. F**, A nano-differential scanning fluorimetry assay was developed to assess the thermal stabilization of recombinant purified human ALDH1A3 by MBE1.5 at two concentrations as compared to melting curves for both native and 2.5% DMSO-treated recombinant ALDH1A3. **G**, Dose-titration specificity analysis of MBE1.5 against a broad collection of human and mouse recombinantly expressed ALDH enzymes. **H**, Competition analysis of varying MBE1.5 concentrations against varying substrate and cofactor concentrations in the ALDH1A3 recombinant assay. Data reported in both untransformed (left) and double reciprocal (right) plots. n= 3 biological replicates per data point. Data are represented as mean ±SEM.

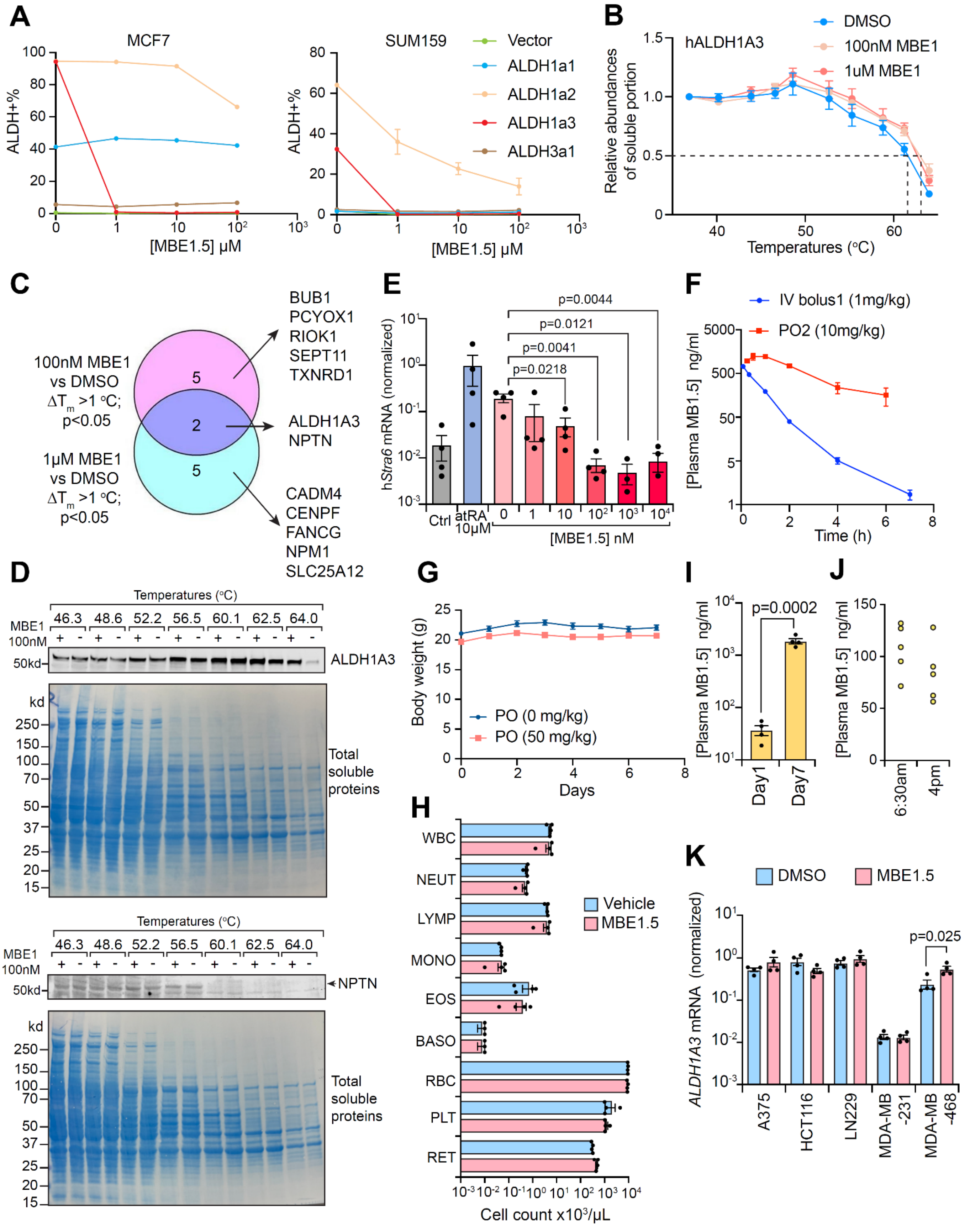
A
MCF7
SUM159
ALDH+%
[MBE1.5] μM
Vector
ALDH1a1
ALDH1a2
ALDH1a3
ALDH3a1
B
hALDH1A3
DMSO
100nM MBE1
1uM MBE1
Relative abundances of soluble portion
Temperatures (°C)
C
100nM MBE1 vs DMSO ΔTm >1 °C; p<0.05
1μM MBE1 vs DMSO ΔTm >1 °C; p<0.05
BUB1
PCYOX1
RIOK1
SEPT11
TXNRD1
ALDH1A3
NPTN
CADM4
CENPF
FANCG
NPM1
SLC25A12
E
hStra6 mRNA (normalized)
p=0.0044
p=0.0121
p=0.0041
p=0.0218
Ctrl
atRA 10μM
[MBE1.5] nM
F
IV bolus1 (1mg/kg)
PO2 (10mg/kg)
[Plasma MB1.5] ng/ml
Time (h)
D
Temperatures (°C)
46.3 48.6 52.2 56.5 60.1 62.5 64.0
MBE1 100nM
ALDH1A3
Total soluble proteins
NPTN
G
Body weight (g)
PO (0 mg/kg)
PO (50 mg/kg)
Days
I
p=0.0002
Day1
Day7
J
6:30am
4pm
H
WBC
NEUT
LYMP
MONO
EOS
BASO
RBC
PLT
RET
Vehicle
MBE1.5
Cell count x10³/μL
K
DMSO
MBE1.5
ALDH1A3 mRNA (normalized)
p=0.025
A375
HCT116
LN229
MDA-MB -231
MDA-MB -468

Figure S5. Specificity, tolerability and pharmacokinetic analysis of MBE1.5 *in vivo*. A, ALDH1A1, ALDH1A2, ALDH1A3 and vector control were stably expressed in MCF7 or SUM159 cells and a dose titration of MBE1.5 was performed against each cell line in the Aldelfuor assay to confirm ALDH1A3 isoform specificity for MBE1.5. n= 3 biological replicates per point. Data are represented as mean ±SEM. **B,** Thermal proteome profiling in A375 cells was performed in DMSO and at two concentrations of MBE1 using a 10 temperature melting curve for isolation, followed by proteomic analysis, filtering and mapping peptide frequency as a composite melting curve, n=3 biological replicates per condition-temperature. **C,** Filtering TPP results by significance ($p<0.05$) and Tm shifts>1 C identified two pools of potential targets stabilized by MBE1, further filtering by a coefficient of variance < 0.1 at both MBE1 concentrations identified ALDH1A3 and NPTN as the only two potential MBE1 thermally-stabilized targets in treated A375 cells. **D**, Soluble proteins were extracted from A375 cells treated with DMSO or 100nM MBE1 after thermal denaturation. ALDH1A3 or NPTN were assessed by WB to confirm the stabilization of ALDH1A3 but not NPTN (55kD, as indicated by arrow) by MBE1. Total protein loading was confirmed by staining on the same blots used to detect either protein. **E,** MBE1.5 was treated at a dose titration in MCF7-ALDH1A3 cells for 24 hours followed by RT-qPCR of *STRA6.* n=3 biological replicates. Student's T-test. Data are represented as mean ±SEM. **F**, *In vivo* pharmacokinetic analysis of MBE1.5 following identification of proper formulations and administration to fasted male CD1 mice at 1 mg/kg intravenous (IV) and 10 mg/kg oral gavage (PO). n = 3 mice per arm. **G-I**, MBE1.5 administered as a suspension to C57BL/6 male mice via bi-daily PO administration for seven days as compared to the vehicle was tracked for body weight (G), complete blood count at day 7 (H) and plasma concentrations of MBE1.5 were analyzed at 12 hours post Day 1 dosing and 12 hours following the last dose (I). n = 4 male mice per arm. **J**, MBE1.5 formulated into mouse diet at 270 ppm was provided to C57BL/6 mice for five days. Upon acclimation, serum MBE1.5 concentrations were calculated following blood draws at 6:30 am and 4 pm. n = 5 mice per arm. **K**, ALDH1A3-positive and negative cell lines were treated with MBE1.5 or 0.1% DMSO for 24 hours and *ALDH1A3* mRNA was calculated following RNA extraction and RT-qPCR. n = 4 biological replicates. Data are represented as mean ±SEM.

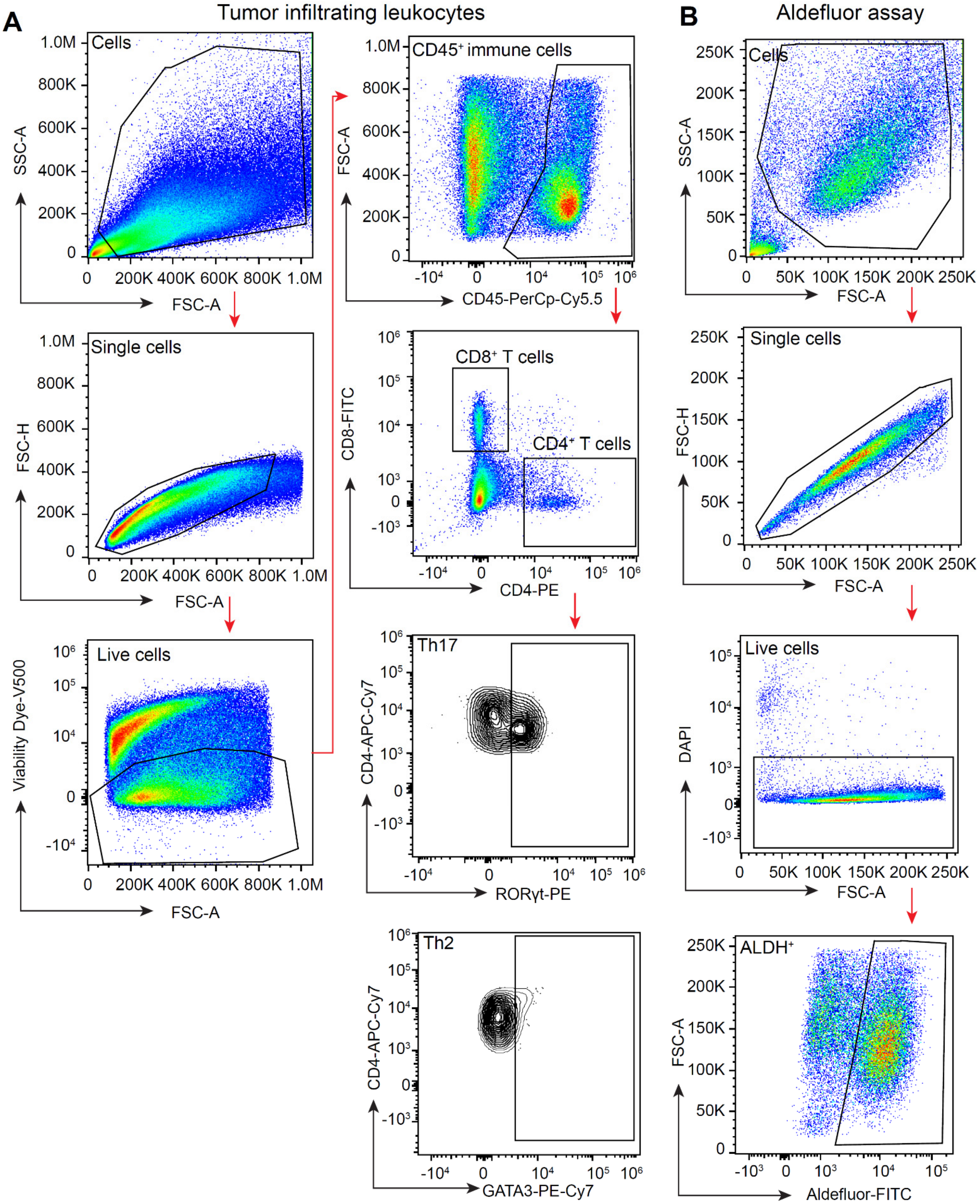
A
Tumor infiltrating leukocytes
B
Aldefluor assay
Cells
SSC-A
FSC-A
Single cells
FSC-H
Live cells
Viability Dye-V500
CD45⁺ immune cells
CD45-PerCp-Cy5.5
CD8⁺ T cells
CD4⁺ T cells
CD8-FITC
CD4-PE
Th17
CD4-APC-Cy7
RORγt-PE
Th2
GATA3-PE-Cy7
DAPI
ALDH⁺
Aldefluor-FITC

Figure S6. Gating strategy for immune profiling and Aldefluor assay analysis by flow cytometry. A, gating strategy for tumor infiltrating immune cells profiling. Dissociated cells were subjected to flow cytometry analysis. Single live $CD45^+$ cells were gated as immune cells, and CD8 vs CD4 are used to differentiate $CD4^+$ vs $CD8^+$ T cells. $CD4^+$ T cell subsets were further characterized by intracellular staining of their transcription factors. RORγt identifies Th17 and GATA3 identifies Th2. **B,** In aldefluor assay, live cells were gated for further analysis. Signal in FITC channel indicates the enzyme activity.

Figure 1D

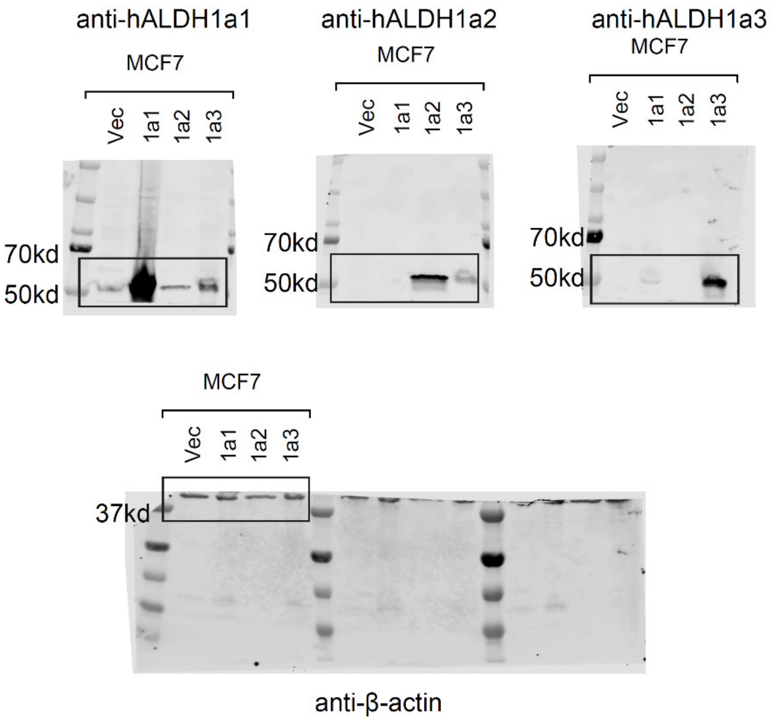

Figure 2B

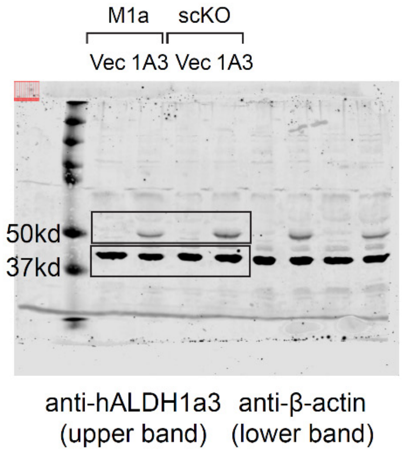

Figure S5D

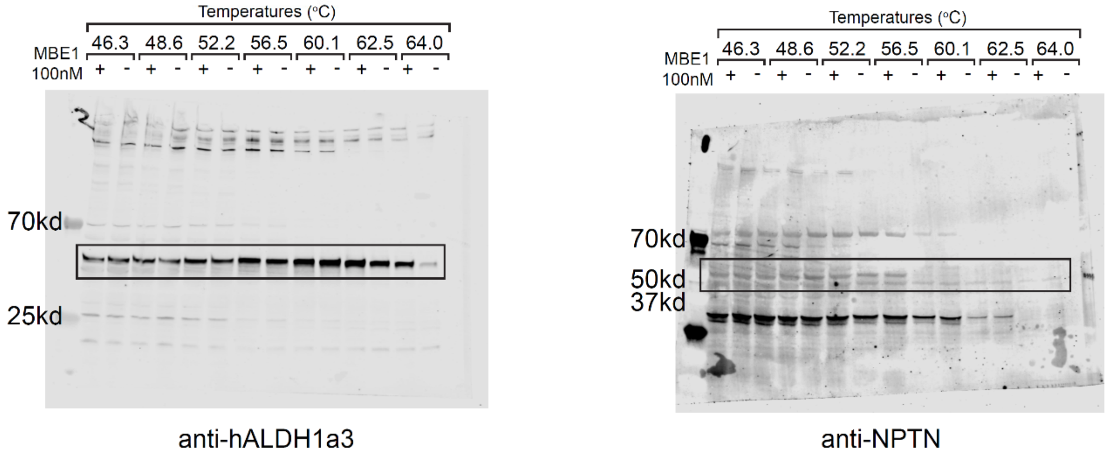

Figure S7. Raw, uncropped western blot scans. Western blot images from the Licor scanner with both colors and molecular weight markers superimposed into one image. Rectangles with text marks the specific regions shown in the main Figures.

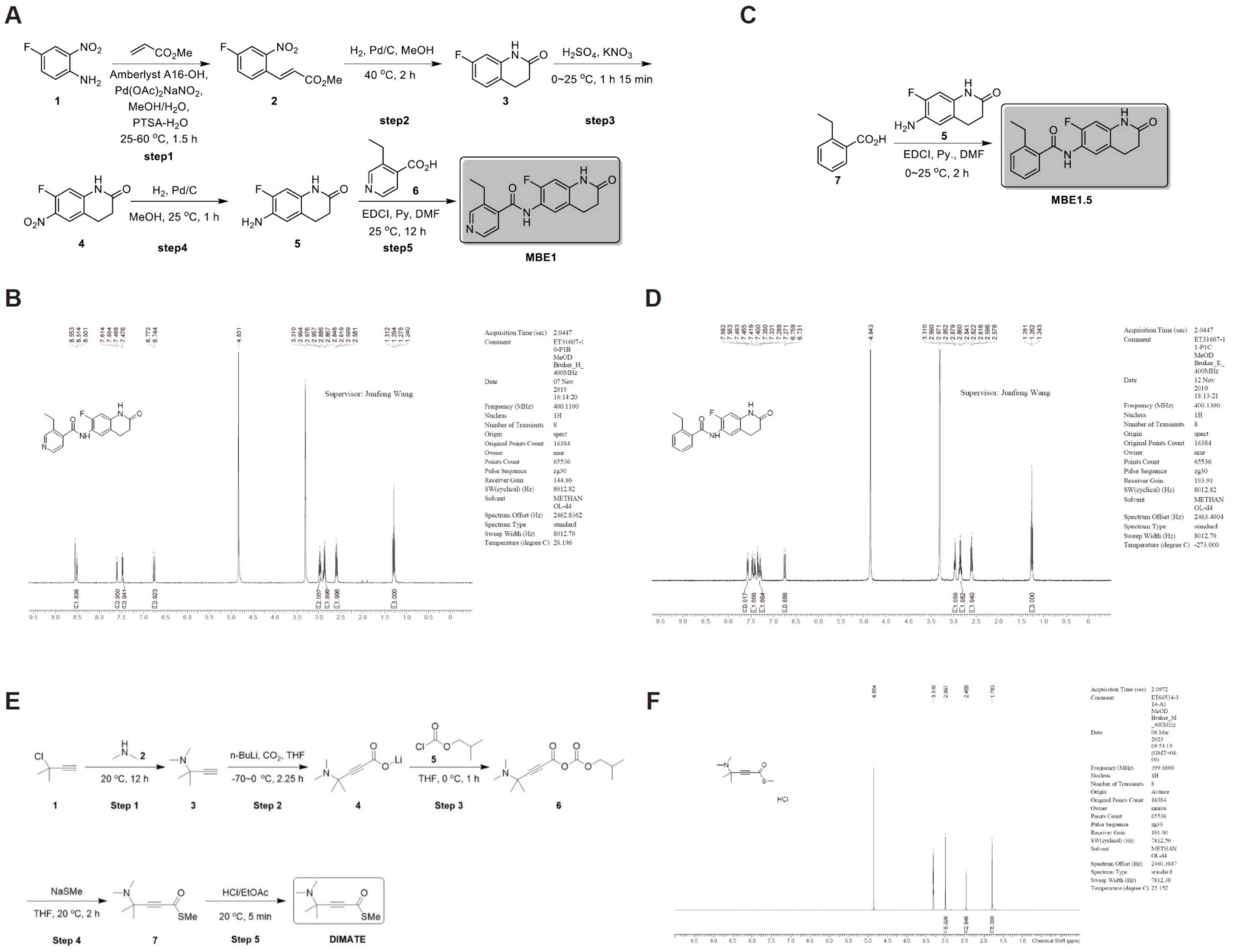

Figure S8. Organic synthesis routes and NMR validation. A-F, Synthetic schema and NMR of molecules MBE1 (A, B), MBE1.5 (C, D), and DIMATE (E, F).
